# MoRgs3 functions in intracellular reactive oxygen species perception-integrated cAMP signaling to promote appressorium formation in *Magnaporthe oryzae*

**DOI:** 10.1128/mbio.00996-24

**Published:** 2024-07-09

**Authors:** Ruiming Zhang, Xinyu Liu, Jiayun Xu, Chen Chen, Zhaoxuan Tang, Chengtong Wu, Xinyue Li, Lei Su, Muxing Liu, Leiyun Yang, Gang Li, Haifeng Zhang, Ping Wang, Zhengguang Zhang

**Affiliations:** 1 Department of Plant Pathology, College of Plant Protection, Nanjing Agricultural University, Nanjing, China; 2 Key Laboratory of Integrated Management of Crop Diseases and Pests, Ministry of Education, Nanjing, China; 3 Department of Microbiology, Immunology, and Parasitology, Louisiana State University Health Sciences Center, New Orleans, Louisiana, USA; Duke University Hospital, Durham, North Carolina, USA

**Keywords:** RGS signaling, ROS sensing, protein phosphorylation, pathogenicity, the blast fungus

## Abstract

Regulator of G-protein signaling (RGS) proteins exhibit GTPase-accelerating protein activities to govern G-protein function. In the rice blast fungus *Magnaporthe oryzae*, there is a family of at least eight RGS and RGS-like proteins (MoRgs1 to MoRgs8), each exhibiting distinct or shared functions in the growth, appressorium formation, and pathogenicity. MoRgs3 recently emerged as one of the crucial regulators that senses intracellular oxidation during appressorium formation. To explore this unique regulatory mechanism of MoRgs3, we identified the nucleoside diphosphate kinase MoNdk1 that interacts with MoRgs3. MoNdk1 phosphorylates MoRgs3 under induced intracellular reactive oxygen species levels, and MoRgs3 phosphorylation is required for appressorium formation and pathogenicity. In addition, we showed that MoRgs3 phosphorylation determines its interaction with MoCrn1, a coronin-like actin-binding protein homolog, which regulates MoRgs3 internalization. Finally, we provided evidence demonstrating that MoRgs3 functions in MoMagA-mediated cAMP signaling to regulate normal appressorium induction. By revealing a novel signal perception mechanism, our studies highlighted the complexity of regulation during the appressorium function and pathogenicity of the blast fungus.

**IMPORTANCE:**

We report that MoRgs3 becomes phosphorylated in an oxidative intracellular environment during the appressorium formation stage. We found that this phosphorylation is carried out by MoNdk1, a nucleoside diphosphate kinase. In addition, this phosphorylation leads to a higher binding affinity between MoRgs3 and MoCrn1, a coronin-like actin-binding protein that was implicated in the endocytic transport of several other RGS proteins of *Magnaporthe oryzae*. We further found that the internalization of MoRgs3 is indispensable for its GTPase-activating protein function toward the Gα subunit MoMagA. Importantly, we characterized how such cellular regulatory events coincide with cAMP signaling-regulated appressorium formation and pathogenicity in the blast fungus. Our studies uncovered a novel intracellular reactive oxygen species signal-transducing mechanism in a model pathogenic fungus with important basic and applied implications.

## INTRODUCTION

The filamentous fungus *Magnaporthe oryzae* causes rice blast that poses the most severe threat to rice production across the world and is also an excellent model organism for studying plant-pathogen interactions (1–3). *M. oryzae* produces the unique infectious structure appressorium following exposure to host-surface cues (4–6). Previous studies have demonstrated that surface signal recognition and appressorium formation are regulated by G-protein and cAMP signaling (7, 8). *M. oryzae* contains three distinct Gα subunit proteins: MoMagA, MoMagB, and MoMagC, as well as a highly conserved cAMP-dependent signaling pathway consisting of the adenylate cyclase MoMac1, the protein kinase A regulatory subunit (Mo)Sum1, and catalytic subunits (Mo)CpkA and (Mo)Cpk2 (7, 9). Additional studies indicated that the non-canonical GPCR Pth11, MoMagA, and RGS proteins MoRgs1 and MoRgs7 mediate the perception of hydrophobic and hard surface cues leading to the activation of the cAMP pathway and normal appressorium formation (10–13).

Recent investigations in *M. oryzae* revealed cellular and molecular insights into the highly conserved signal transduction pathways, including cAMP and mitogen-activated protein (MAP) kinase signaling, governing the appressorium formation process (14–16). Several putative signaling receptors important for appressorium formation have also been identified thus far (8, 17, 18). Previous studies have shown that the RGS protein MoRgs1 plays a critical role in appressorium formation, growth, and virulence (19, 20). MoRgs1 is regulated by casein kinase 2 (MoCk2) through protein phosphorylation, and this regulatory mechanism is indispensable for the GTPase-activating protein (GAP) function of MoRgs1 (18, 20, 21). The RGS-like protein MoRgs7 contains an N-terminal 7-TM typical of G-protein-coupled receptors (GPCR) in addition to the RGS-like domain. Studies showed that MoRgs7 functions in appressorium formation by sensing the environmental hydrophobic cues to participate in the cAMP pathway (22, 23). Similar to MoRgs1 and MoRgs7, the loss of MoRgs3 impaired appressorium formation (19). However, the potential role of MoRgs3 as a novel receptor in sensing environmental cues and its involvement in appressorium formation and virulence remain unknown.

During the asexual life cycle of *M. oryzae*, the conidium produces a germ tube from its tapered end. Upon the perception of specific environmental cues, this germ tube undergoes differentiation, ultimately forming an appressorium (24, 25). These environmental signals encompass factors such as hydrophobicity, surface hardness, leaf waxes, and physicochemical molecules (21, 26). In addition to these environmental factors, intracellular signaling also plays a pivotal role in appressorium formation, involving active substances that mediate signal transduction pathways within the cell upon receptor activation. Examples include intracellular calcium signaling (Ca^2+^), inositol triphosphate (IP3), and reactive oxygen species (ROS) signaling (27, 28). ROS, including hydrogen peroxide (H_2_O_2_), hydroxyl radical (OH^−^), and superoxide (O^2−^), are excited forms of oxygen and toxic byproducts by aerobic metabolism that are important in signaling transduction (29–31). ROS are produced in the metabolic processes of *M. oryzae* and act as signals in a variety of developmental pathways, including appressorium formation. During appressorium morphogenesis, *M. oryzae* accumulates high levels of endogenous ROS in the tips of its germ tubes and immature appressorium (32–34). ROS scavenging delays appressorium differentiation and alters its morphology in *M. oryzae* (35).

As the most important source for ROS generation, the nicotinamide adenine dinucleotide (NADPH) oxidase (NOx) complex orchestrates the transfer of electrons from NADPH to the plasma membrane, catalyzing the reduction of O_2_ to produce superoxide O_2_. Subsequently, superoxide dismutase catalyzes the conversion of superoxide O_2_ into H_2_O_2_ (36). These processes are important for cell proliferation, signaling, and apoptosis. Three NADPH oxidases, MoNox1, MoNox2, and MoNox3, involved in ROS biosynthesis were identified in *M. oryzae* (36). MoNox1 and MoNox2 are important for ROS accumulation during appressorium maturation and formation of penetration pegs (36, 37). However, the oxidative burst reaction occurs rapidly; the pathogen must quickly counteract this stress. Plant pathogens have evolved various strategies to rapidly detoxify ROS through cellular enzymatic and non-enzymatic mechanisms (38, 39). Previous studies have found that thioredoxin MoTrx2 is involved in the ROS-detoxification process regulated by the transcription factor MoAp1. *MoTRX2* deletion leads to limited intracellular ROS clearance and excessive oxidation of the intracellular environment that affects appressorium formation (40). Recent research showed that the *M. oryzae* nucleoside diphosphate kinase homolog MoNdk1 mediates redox balance by regulating upstream intracellular nucleotide pools, and MoNdk1 participates in the detoxification process of intracellular ROS (41–43). The loss of Δ*Mondk1* resulting in heightened oxidative stress, increased ROS generation, altered G protein signaling regulation, and aberrant appressorium formation demonstrated that MoNdk1 is a key player in maintaining redox balance (44). Such evidence indicated the an intricate physiological balance between the timely biogenesis and ROS detoxification plays an important role in the formation and function of the appressorium in *M. oryzae*.

We sought to understand the precise impact of an oxidative environment on appressorium formation and virulence and MoRgs3 regulatory mechanisms in *M. oryzae*. We found that MoRgs3 is subject to MoNdk1 phosphorylation in response to ROS signals. MoNdk1 modulates MoRgs3-MoNdk1 binding affinity through the actin-binding Coronin-like protein MoCrn1, and MoNdk1 and MoCrn1 participate in the transportation process of MoRgs3. These concordant cellular processes ensure a critical role of G-protein/cAMP-PKA signaling in regulating the growth, appressorium formation, and pathogenicity of *M. oryzae*.

## RESULTS

### MoRgs3 is phosphorylated during appressorium formation

**Fig 1:**
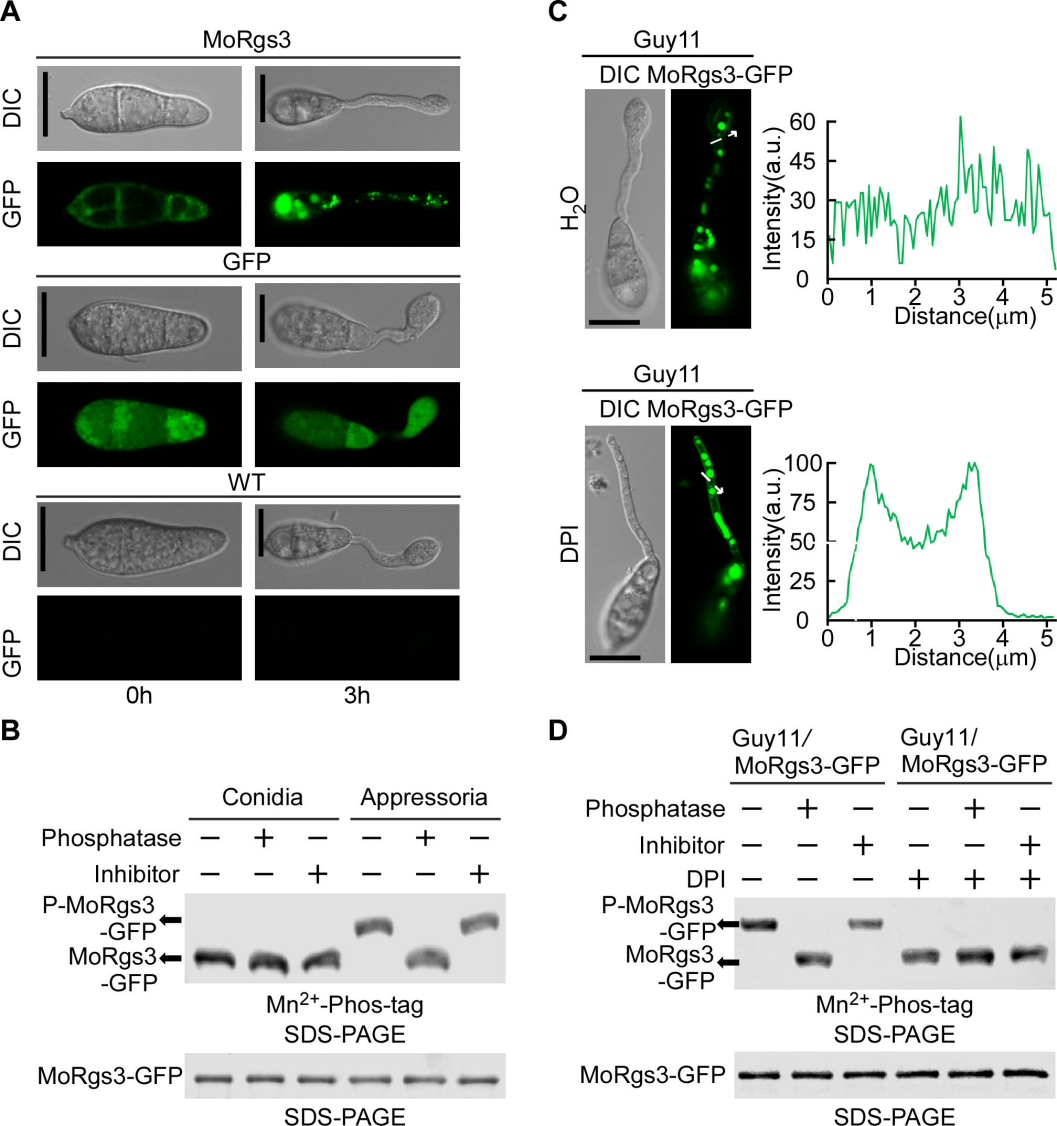
Phosphorylation dynamics of MoRgs3 during early appressorium development in *M. oryzae*. (**A**) For MoRgs3-GFP observations, conidia were inoculated on plastic coverslips and incubated in a moist chamber. DIC and epifluorescence images were captured at the indicated time points. Scale bar: 5 µm. (**B**) MoRgs3-GFP proteins were extracted from transformants at conidia and appressorium stages, then treated with phosphatase and phosphatase inhibitors. and shifted by Mn^2+^-Phos-tag SDS-PAGE and normal SDS-PAGE with the anti-GFP antibody. For total protein extraction, 1 mL lysis buffer [10 mM Tris-HCl, pH7.5, 150 mM NaCl, 0.5 mM EDTA, 0.5% NP-40 (Sigma-Aldrich, I3021)] with 2 mM phenyl methyl sulfonyl fluoride (PMSF) (Beyotime Biotechnology, ST506-2), proteinase inhibitor cocktail (Sigma-Aldrich, 11836170001), and deacetylation inhibitors [50 mM nicotinamide, 50 mM sodium butyrate, 5 mM Trichostatin A (Sigma-Aldrich, T1952)] were used to resuspend ground powder of appressorium and conidia. (**C**) For MoRgs3-GFP observations, conidia were inoculated on plastic coverslips and incubated in a moist chamber. DIC and fluorescence images were collected 3 hours after conidial germination. Inhibition of *M. oryzae* superoxide production by NADPH oxidase inhibitor diphenylene iodonium (DPI). The appressorium was soaked in 25 µM DPI solution for 20 min before DIC, and fluorescence images were collected. Scale bar: 5 µm. (**D**) The phosphorylation *in vivo* was determined. MoRgs3-GFP protein was extracted from transformants of Guy11/MoRgs3-GFP strains treated with or without 25 µM DPI inhibitors and then treated with phosphatase and phosphatase inhibitors and shifted by Mn2+-Phos-tag SDS-PAGE and normal SDS-PAGE with anti-GFP antibody.

Previous studies have shown that MoRgs3 is required for normal appressorium formation in *M. oryzae* (19). To examine this functional role, we first generated a MoRgs3-GFP fusion construct and introduced it into the Δ*Morgs3* strain. Subcellular localization studies indicated that MoRgs3 is mainly localized in the plasma membrane (PM) at the conidial stage but distributed in the cytoplasm at the appressorial stage (Fig. 1A). To verify the above results, we used the membrane dye FM4-64 and vesicle dye CMAC to localize MoRgs3 in the Δ*Morgs3*/*MoRGS3* strain. The fluorescence micrograph showed that MoRgs3-GFP co-localized with FM4-64 in the PM of conidia, while MoRgs3-GFP co-localized with CMAC in the intracellular vesicle during appressorium formation (Fig. S1A).

As phosphorylation of RGS proteins, such as MoRgs7, is important in their cellular localization (22), we investigated MoRgs3 phosphorylation using Mn^2+^-Phos-tag gel electrophoresis analysis. Cell extracts were obtained from conidial and appressorial stages of a MoRgs3-GFP transformant strain treated with or without a phosphatase or a phosphatase inhibitor. The results showed that the protein band representing the phosphorylated MoRgs3-GFP (p-MoRgs3) is apparent in the appressorial stage but not the conidial stage. The p-MoRgs3 band was absent in the presence of a phosphatase but more prominent in the presence of a phosphatase inhibitor (Fig. 1B). The above observation indicated that the changes of MoRgs3 localization at different periods are linked to its phosphorylation status and MoRgs3 is phosphorylated during the appressorial stage.

### Phosphorylation of MoRgs3 is influenced by intracellular ROS

MoRgs1 and MoRgs7 undergo protein phosphorylation following the perception of hydrophobic surface cues required for appressorium formation in *M. oryzae* (22). To test whether MoRgs3 has a similar process, we examined MoRgs3 phosphorylation in response to hydrophobic surface cues by comparing proteins extracted from germinated conidia after 4 hours of incubation on hydrophobic and hydrophilic surfaces. The results showed that MoRgs3-GFP is phosphorylated at both interfaces (Fig. S1B), suggesting that MoRgs3 phosphorylation may not be induced by hydrophobicity. In addition to sensing hydrophobic surface cues, ROS burst during germination is also important for the formation of the appressorium, which is a process inhibited by the Nox inhibitor DPI (35, 45, 46). We examined whether MoRgs3 phosphorylation responds to changes in intracellular ROS levels. Indeed, MoRgs3 remained on the PM following DPI treatment suggesting that MoRgs3 phosphorylation is ROS dependent (Fig. 1C and D).

In *M. oryzae*, redox homeostasis is tightly balanced by multiple intracellular ROS-metabolizing processes. Previous studies have shown that MoTrx2 functions downstream of the MAP kinase signaling pathway and it mediates a redox system common in eukaryotic cells. MoTrx2 catalyzes various redox reactions through reversible redox activities and controls intracellular ROS levels by modifying the redox state of key signaling components (39, 40). Previous studies also showed that the Δ*Motrx2* mutant displays increased intracellular ROS levels during conidial germination (40). We thus transferred MoRgs3-GFP into the Δ*Motrx2* mutant and found that almost all MoRgs3-GFP was localized to the endosomes of the mutant during the conidial stage in contrast to PM localization in the wild-type Guy11 strain (Fig. S1D and F). This localization was inhibited by DPI (Fig. S1E and G). We also observed the localization of MoRgs3-GFP at 3 hours post-inoculation, and the Δ*Motrx2* mutant displayed MoRgs3-GFP at the endosomes of the germ tube (Fig. S2A). Retention of MoRgs3-GFP in the PM was also observed when germinated conidia were treated with DPI (Fig. S2B). These results indicated that intracellular ROS play a key role in regulating localization of MoRgs3.

We further found that MoRgs3 can be phosphorylated in high intracellular ROS levels, such as at the appressorium stage of Guy11 or in the Δ*Motrx2* mutant strain. Phosphorylation disappeared following DPI treatment (Fig. S1C and S2C). We concluded that an increased ROS level and an oxidized intracellular environment promote MoRgs3 phosphorylation.

### MoRgs3 is phosphorylated by MoNdk1

**Fig 2:**
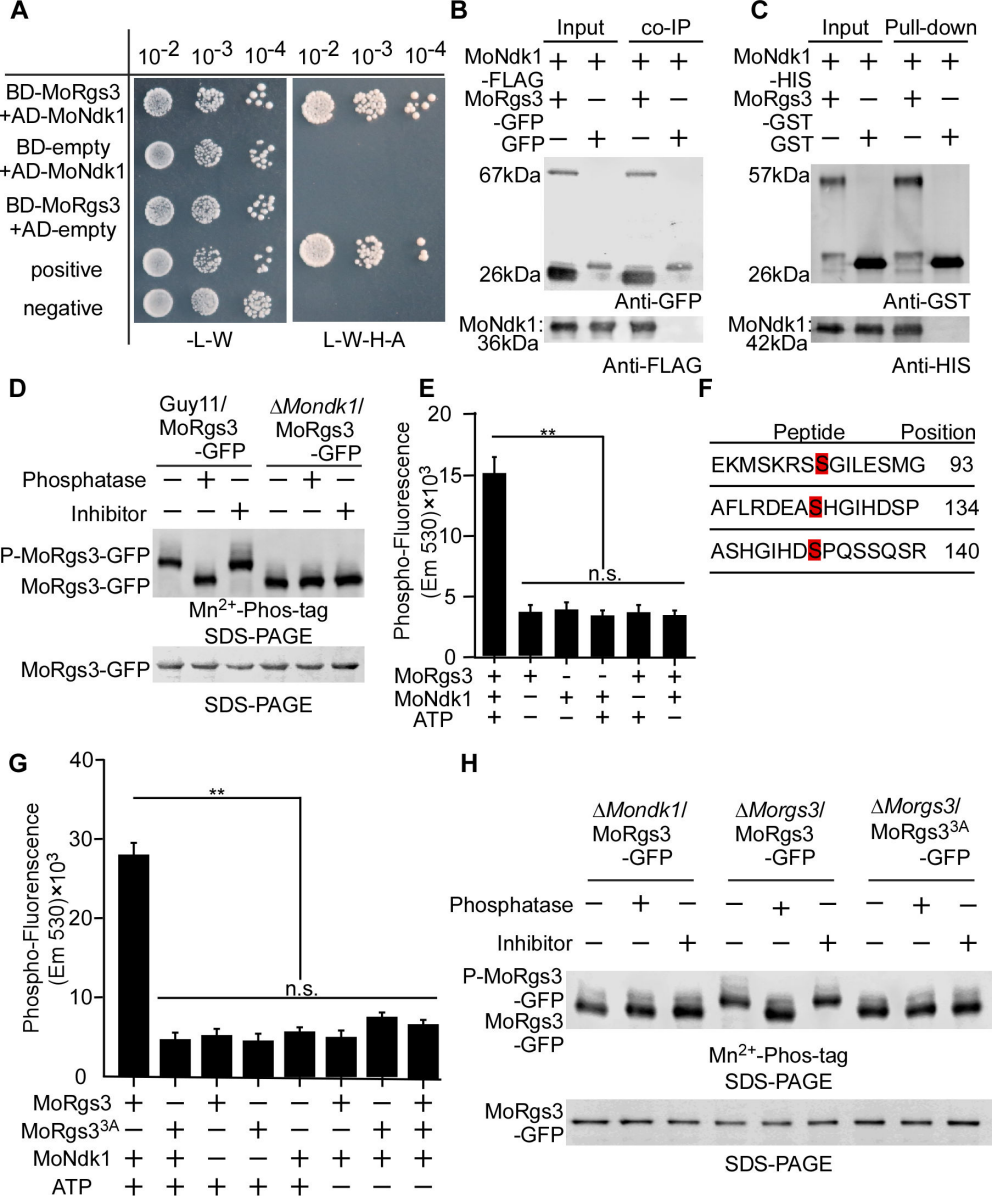
MoNdk1 functions as a kinase to phosphorylate MoRgs3. (**A**) Y2H assays for examining interactions between AD-MoRgs3 and BD-MoNdk1. Yeast co-transformants expressing the bait and prey constructs were isolated on SD-Leu-Trp plate for 3 days and screened by culturing on SD-Ade-His-Leu-Trp plates for 5 days. (**B**) Co-IP assays for the interaction between MoRgs3-GFP and MoNdk1-Flag transformants were introduced into empty-GFP as a control. Total proteins were extracted individually as the total proteins, eluted from the anti-GFP agarose beads, and analyzed by western blot with the anti-GFP or anti-FLAG antibodies. (**C**) The interaction between GST-MoRgs3 and His-MoNdk1 was conducted by GST pull-down assays. GST-MoRgs3, His-MoNdk1, and GST were expressed and purified by affinity chromatography. Bound proteins were separated by SDS-PAGE in duplicate and analyzed by western blot with the anti-HIS (Mouse; M20001; Abmart) and anti-GST antibodies (Mouse; M20007; Abmart). (**D**) Phosphorylation analysis of MoRgs3 *in vivo* by Mn^2+^-Phos-tag gel. MoRgs3-GFP proteins treated with phosphatase and phosphatase inhibitors were detected by the GFP antibody and shifted by Mn^2+^-Phos-tag SDS-PAGE and normal SDS-PAGE, respectively. (**E**) Phosphorylation analysis *in vitro* by the fluorescence detection in tube (FDIT) method. Purified proteins of GST-MoNdk1 and His-MoRgs3 were constructed for protein kinase reactions in the presence of ATP. The fluorescence signal was measured in a microplate reader. Asterisks denote statistically significant difference according to ANOVA (**P < 0.01, *n* = 3). Values are means of three replications and standard deviation (SD). (**F**) Identification of differentiated phosphorylation sites in the wild type strain Guy11 compared with Δ*Mondk1* strains by LC-MS-MS (**Q-E**). (**G**) Phosphorylation analysis by the FDIT method *in vitro*. GST-MoRgs3, GST-MoRgs3^3A^ (S93A, S134A, S140A), His-MoNdk1, were constructed for protein kinase reaction assays in the presence of ATP and a kinase reaction buffer. Fluorescence was measured in a microplate reader. Fluorescence at 590 nm (excited at 530 nm) was measured. Values are means of three replications and SD (**P < 0.01, *n* = 3). (**H**) Phosphorylation analysis of MoRgs3 and site-directed mutagenesis MoRgs3^3A^ and MoRgs3^3D^ *in vivo*. Proteins were extracted from corresponding transformants and treated with phosphatase and phosphatase inhibitors, then detected by the GFP antibody, and shifted by Mn^2+^-Phos-tag SDS-PAGE and normal SDS-PAGE, respectively. All experiments were conducted with three biological repetitions and three replicates.

To identify proteins that bind to and potentially phosphorylate MoRgs3, we performed a yeast two-hybrid (Y2H) screen to first identify MoRgs3-interacting proteins. Using MoRgs3-BD as a bait, we screened a cDNA activator library of *M. oryzae*. Following gene sequencing analysis of several potential binding partners, we identified MoNdk1, a ribonucleoside diphosphate kinase, encoded by the gene locus MGG_08622 (S1 Text). We further verified the MoRgs3-MoNdk1 interaction by *in vitro* Y2H, GST pull-down, and *in vivo* co-IP and split YFP BiFC assays (Fig. 2A through C; Fig. S3D). Moreover, the BiFC assay showed that fluorescence is located at the inner PM and dynamic tubule-vesicular compartments (Fig. S3D). We then performed *in vivo* phosphorylation experiments that showed MoRgs3 could not be phosphorylated in the absence of MoNdk1 (Fig. 2D). This finding was confirmed by an *in vitro* phosphorylation assay using a protein gel-staining fluorescence dye as described previously (47). Co-incubation of purified MoRgs3 and MoNdk1 proteins generated significantly higher phospho-fluorescence than the control (Fig. 2E). The results concluded that MoRgs3 is a phosphorylation substrate of MoNdk1.

To identify the phosphorylation sites of MoRgs3 by MoNdk1, we purified MoRgs3 from Δ*Morgs3/MoRgs3-GFP* and Δ*Mondk1/MoRgs3-GFP* strains and performed liquid chromatography-tandem mass spectrometry analysis. Three serine phosphorylation sites, S93, S134, and S140, were identified (Fig. 2F; Fig. S3A through C). To test if these three residues are MoNdk1-dependent phosphorylation sites, we generated three serine (S) to alanine (A) and aspartic acid (D) site-directed mutagenesis constructs fused with either GFP or GST to mimic the sustainable unphosphorylated (MoRgs3^3A^) and phosphomimic (MoRgs3^3D^) status. *In vivo* Mn^2+^-Phos-tag gel analysis and *in vitro* FDIT phosphorylation assays confirmed that all three residues are phosphorylated by MoNdk1 (Fig. 2G and H). Y2H confirmed that only MoRgs3 and MoRgs3^3A^ interact with MoNdk1, but not MoRgs3^3D^. These results also showed that the phosphorylated MoRgs3 is dissociated from MoNdk1 (Fig. S3E).

### ROS contributes to MoNdk1-dependent phosphorylation of MoRgs3

Previous studies showed the Δ*Mondk1* mutant has an impaired cellular redox status (44). To assess the physiological consequence of perturbed cellular redox homeostasis in the Δ*Mondk1* mutant strain, we examined ROS production during conidial germination and appressorium formation using nitroblue tetrazolium, a compound that forms a dark-blue water-insoluble formazan precipitate upon superoxide radical reduction (29). We detected superoxide production in conidia as early as 20 min after inoculation (Fig. S4G). Notably, formazan precipitates were more intense in the Δ*Motrx2* and Δ*Mondk1* strains, although lighter staining was seen in all conidia.

**Fig 3:**
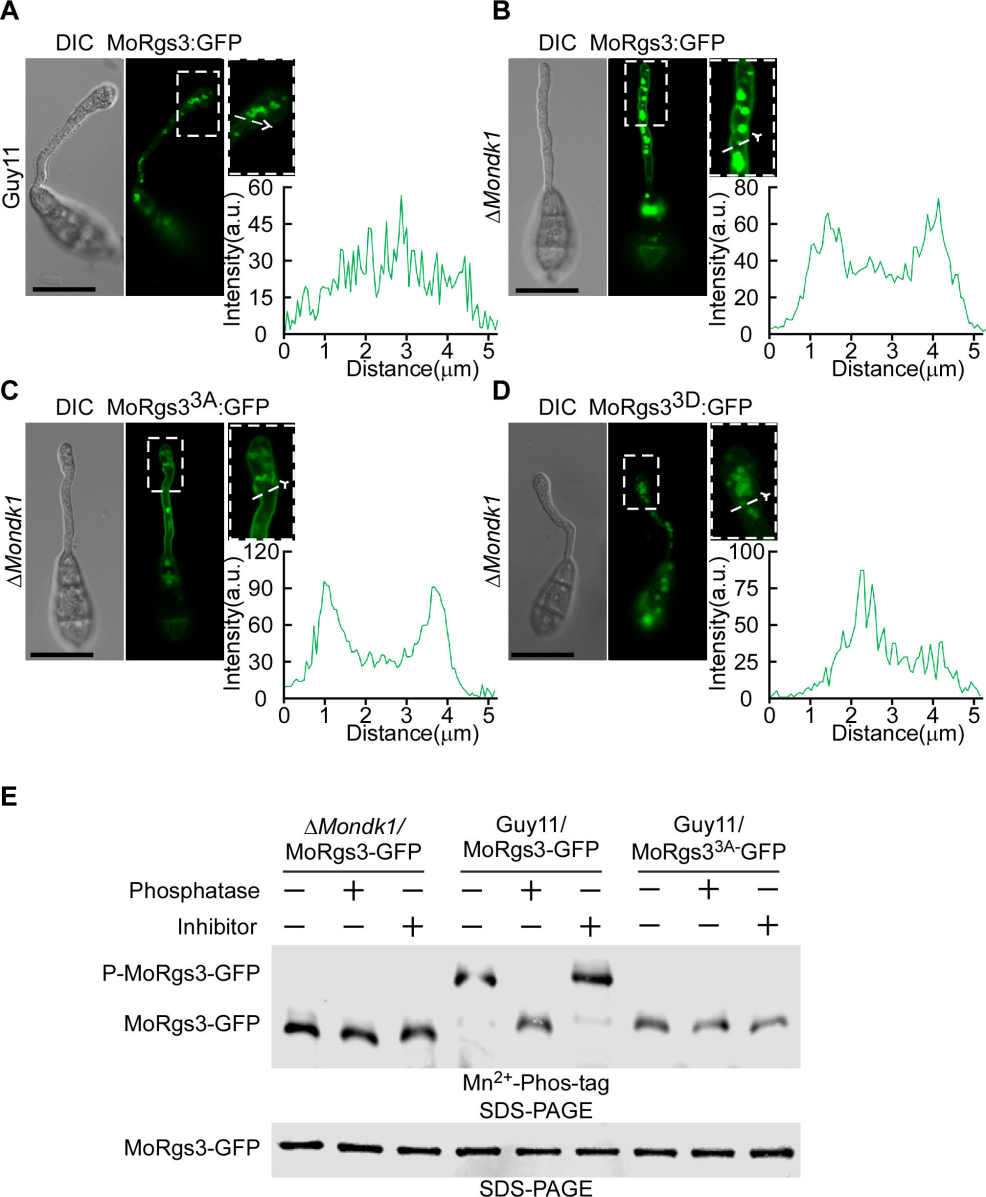
MoNdk1-dependent MoRgs3 phosphorylation is important for MoRgs3 localization. (**A**) MoRgs3-GFP is localized in the PM and the endosome during the appressorial stage. (**B AND C**) The signal of MoRgs3-GFP was enhanced at the PM of germ tubes on the hydrophobic surface at 3 hpi when MoRgs3 was sustainably unphosphorylated. (**D**) State of activation MoRgs3^3D^-GFP is localized in the endosome during the appressorial stage. The magnified region pointed by white arrows was conducted with line scan analysis. Percentage of a pattern showed in image was calculated by observation for 100 germinated conidia that were randomly chosen. This experiment was performed with three biological replicates. Scale bar: 5 µm. (**E**) *In vivo* phosphorylation analysis of MoRgs3-GFP in Δ*Mondk1* and wild-type Guy11.

A previous study suggested that NDK enzymes impact the signal complex assembly at the PM via caveolae formation (33). We subsequently found that MoNdk1 and MoRgs3 interact and co-locate in both the inner PM and dynamic tubulo-vesicular compartments, similar to MoRgs3 (Fig. S3D). We hypothesized that MoRgs3 phosphorylation by MoNdk1 could impact this localization pattern. To test this hypothesis, we observed the spatial distribution of MoRgs3-GFP, MoRgs3^3A^-GFP, and MoRgs3^3D^-GFP in germinated conidia on the hydrophobic surface at 3 hours post-inoculation. There was a higher MoRgs3-GFP fluorescence accumulated at the PM of the germ tube in the Δ*Mondk1* mutant when compared with Guy11 (Fig. 3A and B). We also found that MoRgs3^3A^-GFP fluorescence is enhanced at the PM, which was similar to MoRgs3-GFP in the Δ*Mondk1* mutant but in contrast to MoRgs3^3D^-GFP (Fig. 3C and D). This finding indicated that MoNdk1-mediated phosphorylation of MoRgs3 alters the localization and affects the function of MoRgs3. These results were confirmed by *in vivo* phosphorylation of MoRgs3 and MoRgs3^3A^ using Mn^2+^ Phos-tag gel (Fig. 3E).

We next tested whether MoRgs3 phosphorylation by MoNdk1 is also ROS dependent. We generated a Δ*Mondk1* Δ*Motrx2* strain and introduced MoRgs3-GFP into this double-knockout mutant. At the conidial stage, the Δ*Mondk1* Δ*Motrx2* mutant exhibited less MoRgs3-GFP at the endosomes compared with Δ*Motrx2* (Fig. S4A and B). After 3 hpi, Δ*Mondk1* Δ*Motrx2* showed the phosphorylation of MoRgs3, primarily concentrated in the PM, in comparison to the Δ*Motrx2* strain with a more oxidative intracellular environment (Fig. S4C and D). Phosphorylation analysis indicated that MoRgs3 phosphorylation requires the combined action of intracellular reactive oxygen species and MoNdk1 (Fig. S4 Eand F). These results collectively suggested that MoNdk1 may function as a sensor for ROS-dependent MoRgs3 phosphorylation.

### MoRgs3 phosphorylation is required for appressorium formation and pathogenicity

**Fig 4:**
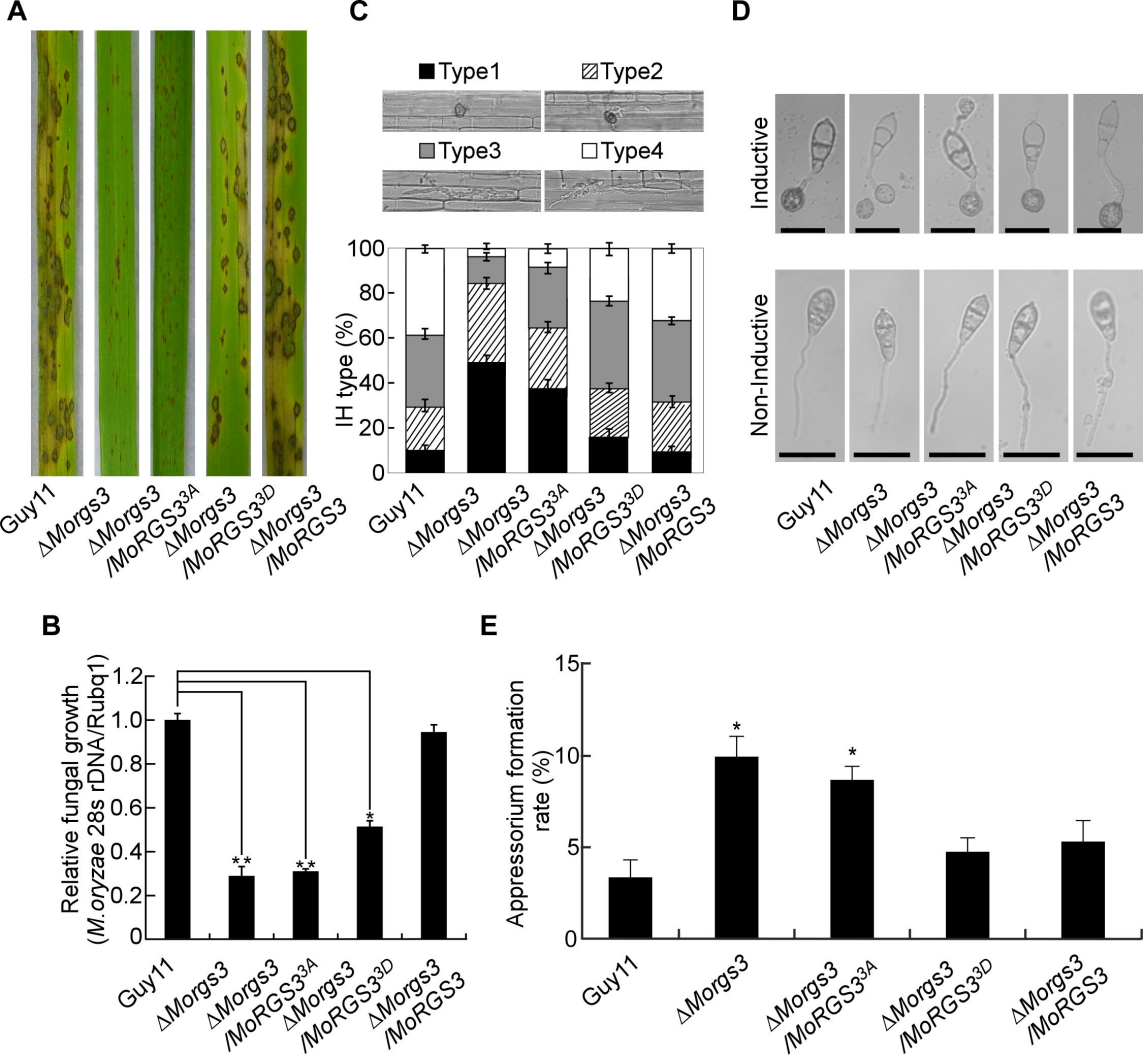
Activating MoRgs3 phosphorylation restores the deficiency of appressorium formation and pathogenicity to the Δ*Morgs3* mutant. (**A**) Pathogenicity assay was conducted with 2-week-old rice seedlings (*Oryza sativa* cv. CO39), which were sprayed by conidial suspensions (5 × 10^4^ spores/mL) of each strain. After 7 days post-incubation (dpi), diseased leaves were photographed. (**B**) Diseased leaf areas were evaluated by quantitative analysis. (**C**) Detailed observation and statistical analysis of infectious hyphal type in rice sheath cells at 36 hpi. (type 1, appressorium formation with no penetration; type 2, penetration with short IH; type 3, invasive IH extended within a plant cell; type 4, extensive hyphal growth). One hundred infectious hyphae were assessed for each strain. The mean values of three repeated experiments with standard deviations are shown. Scale bar: 10 µm. (**D**) Appressorium formation assays. The conidia of each strain were photographed after 24 hours of incubation. Scale bar: 5 µm. (**E**) Percentage of two appressoria, indicating its defect in appressorium development. One hundred conidia of each strain were observed after 24 hours of incubation. Error bars represent SD, and asterisks represent significant differences (*P < 0.05).

To examine if MoRgs3 phosphorylation is required for appressorium formation and virulence, conidia of Guy11, the Δ*Morgs3* mutant, and the complemented Δ*Morgs3/MoRGS3* strains, as well as site-directed Δ*Morgs3/MoRGS3^3A^* and Δ*Morgs3/MoRGS3^3D^* mutant strains, were sprayed on susceptible rice seedlings (CO-39). Very few lesions were found in leaves infected with Δ*Morgs3* and Δ*Morgs3/MoRGS3^3A^* in comparison to Δ*Morgs3/MoRGS3^3D^* and other strains (Fig. 4A and B). Rice sheath penetration assays were also conducted by observing 100 appressoria per strain and categorizing invasive hyphae (IH) types as previously described (19). Over 50% of Δ*Morgs3* and Δ*Morgs3/MoRGS3^3A^* appressoria was defective in penetration and 40% of appressoria penetrated but formed less extended IH. In contrast, 75% of appressoria in Guy11 successfully penetrated rice cells with about 40% producing strong IH (Fig. 4C). These results indicated that MoNdk1-dependent MoRgs3 phosphorylation is critical for the appressorial formation and pathogenicity of *M. oryzae*.

Due to the importance of the G-protein signaling pathway during the early stages of appressorium formation, we examined the effect of MoRgs3 phosphorylation on appressorium formation and morphology. We found that the effect of MoRgs3 phosphorylation in appressorial formation was consistent with the defect of MoRgs1 and MoRgs7. Abnormal perception of hydrophobic cues resulted in delayed appressorium formation and abnormal appressorium morphology, including the formation of two appressoria from a single conidium (19). Indeed, approximately 10% of conidia formed double appressoria in the Δ*Morgs3* and Δ*Morgs3/MoRGS3^3A^*, while lower than 5% did so in Δ*Morgs3/MoRGS3^3D^* (Fig. 4D and E). The difference of appressorium formation rates on hydrophobic slides was also analyzed. The results showed that appressorium formation is significantly decreased in Δ*Morgs3* and Δ*Morgs3/MoRGS3^3A^* in comparison with Guy11 and the complemented strains (Fig. S5A and B ).

MoNdk1 was previously reported to be required for pathogenicity (44). To examine whether such a role is relevant to MoRgs3 phosphorylation, we expressed MoRgs3^3D^-GFP constructs into Δ*Mondk1* and examined its vegetative growth, conidiation, and virulence. Less than 35% appressoria was functional in the Δ*Morgs3* and Δ*Mondk1/MoRGS3^3A^* strains at 24 hpi, in contrast to 60% in Δ*Mondk1/MoRGS3^3D^*. The results showed that continuous phosphorylation of MoRgs3 partial restores the defect of Δ*Mondk1* in growth and virulence (Fig. S5C and D ). These results showed that MoRgs3 phosphorylation plays an important role in the pathogenesis of *M. oryzae*.

### Phosphorylation is vital for MoRgs3 internalization during appressorium development

**Fig 5:**
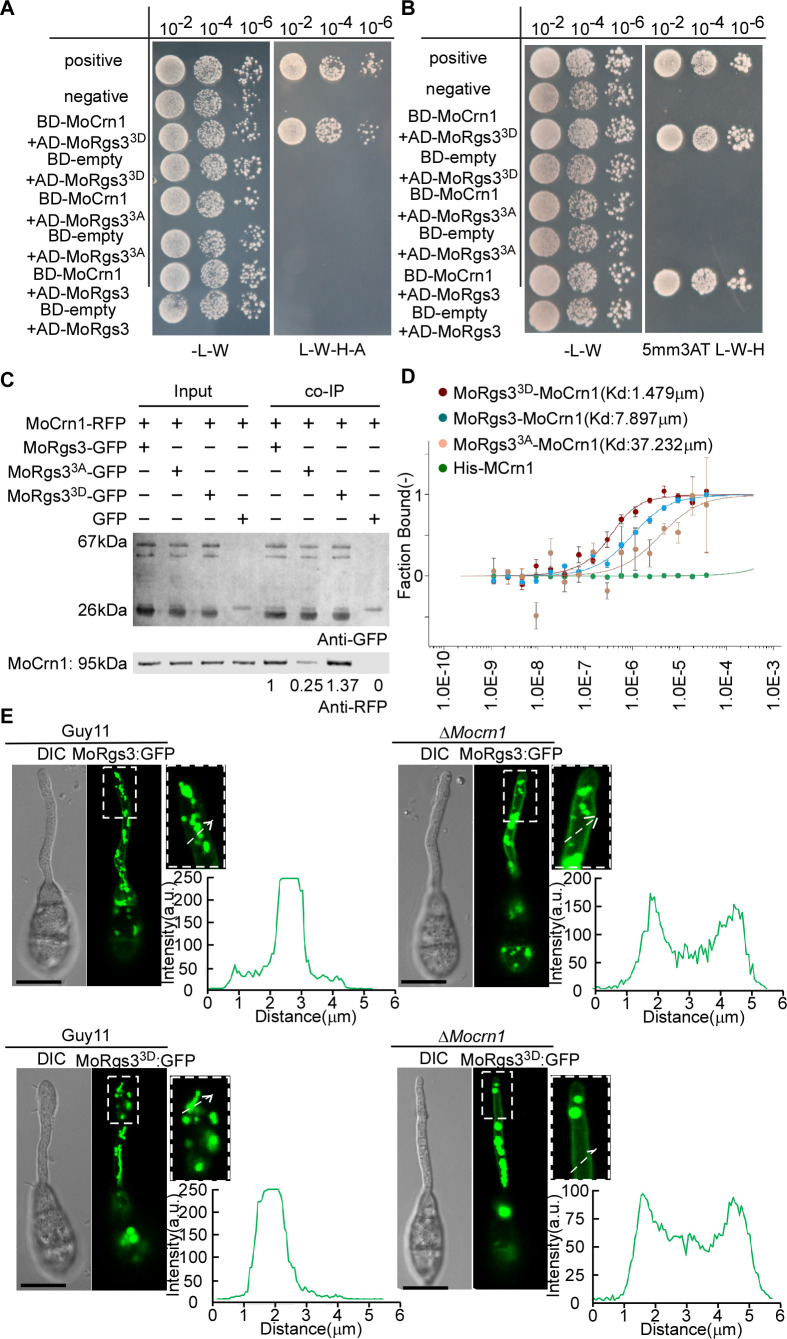
MoRgs3 interacts with MoCrn1. (**A and B**) Yeast two-hybrid analysis. MoCrn1 was co-introduced with MoRgs3 and its site-directed mutagenesis MoRgs3^3A^ and MoRgs3^3D^ into the AH109 strain, respectively. Transformants were plated on SD-Leu-Trp (as control), SD-His-Leu-Trp (for initial selection), and SD-Leu-Trp-His-Ade (for further selection) for 5 days. (**C**) Co-IP assays among all three states of MoRgs3 (MoRgs3, MoRgs3^3A^, and MoRgs3^3D^) and MoCrn1-RFP. Empty GFP transformants introduced by all three states of the RGS3 (MoRgs3, MoRgs3^3A^, and MoRgs3^3D^) were the controls. Total proteins were extracted individually as the total proteins, then eluted from the anti-RFP agarose beads, and analyzed by western blot with corresponding antibodies. ImageJ was used to analyze and compare the gray values of RFP co-IP results. (**D**) MST showing binding properties of MoRgs7 with MoCrn1. Sorting by Kd value, MoRgs3^3D^<MoRgs3<MoRgs3^3A^. Kd, dissociation constant. (**E**) At 3 hours after conidial germination, persistently phosphorylated MoRGS3^3D^-GFP in Guy11 was predominantly localized in the endosome of the germ tube and MoRGS3^3D^-GFP in Δ*MoCrn1* was predominantly localized in the PM. The magnified region pointed by white arrows was conducted with line scan analysis. Percentage of a pattern showed in image was calculated by observation for 100 germinated conidia that were randomly chosen. This experiment was performed with three biological replicates. Scale bar: 5 µm.

We have previously shown that MoRgs1 and MoRgs7 functioning in appressorium formation involve their active entry into the endosome and that the endocytic transport is assisted by the coronin-like actin-binding protein, MoCrn1 (20, 22). We then tested if MoRgs3 has a similar functional mechanism. We found that MoRgs3 can be localized to the late endosomes which are the main components of the endocytic pathway (Fig. S6D). MoRgs3 has three transmembrane domains, in addition to the RGS domain. An Y2H experiment showed that the MoRgs3 RGS domain could interact with MoCrn1 (Fig. S6A and B ). To further test if MoRgs3 phosphorylation affects this binding, we assessed the interactions of MoRgs3, MoRgs3^3A^, and MoRgs3^3D^ with MoCrn1 by Y2H, pull-down, *in vivo* co-IP, and MST assays (Fig. 5A through D and 6C). The results showed that MoRgs3 phosphorylation is critical for its binding with MoCrn1. The sustainable phosphomimic MoRgs3 (MoRgs3^3D^) had a high affinity for MoCrn1, while the sustainable unphosphorylated MoRgs3 (MoRgs3^3A^) interacted weakly with MoCrn1.

**Fig 6:**
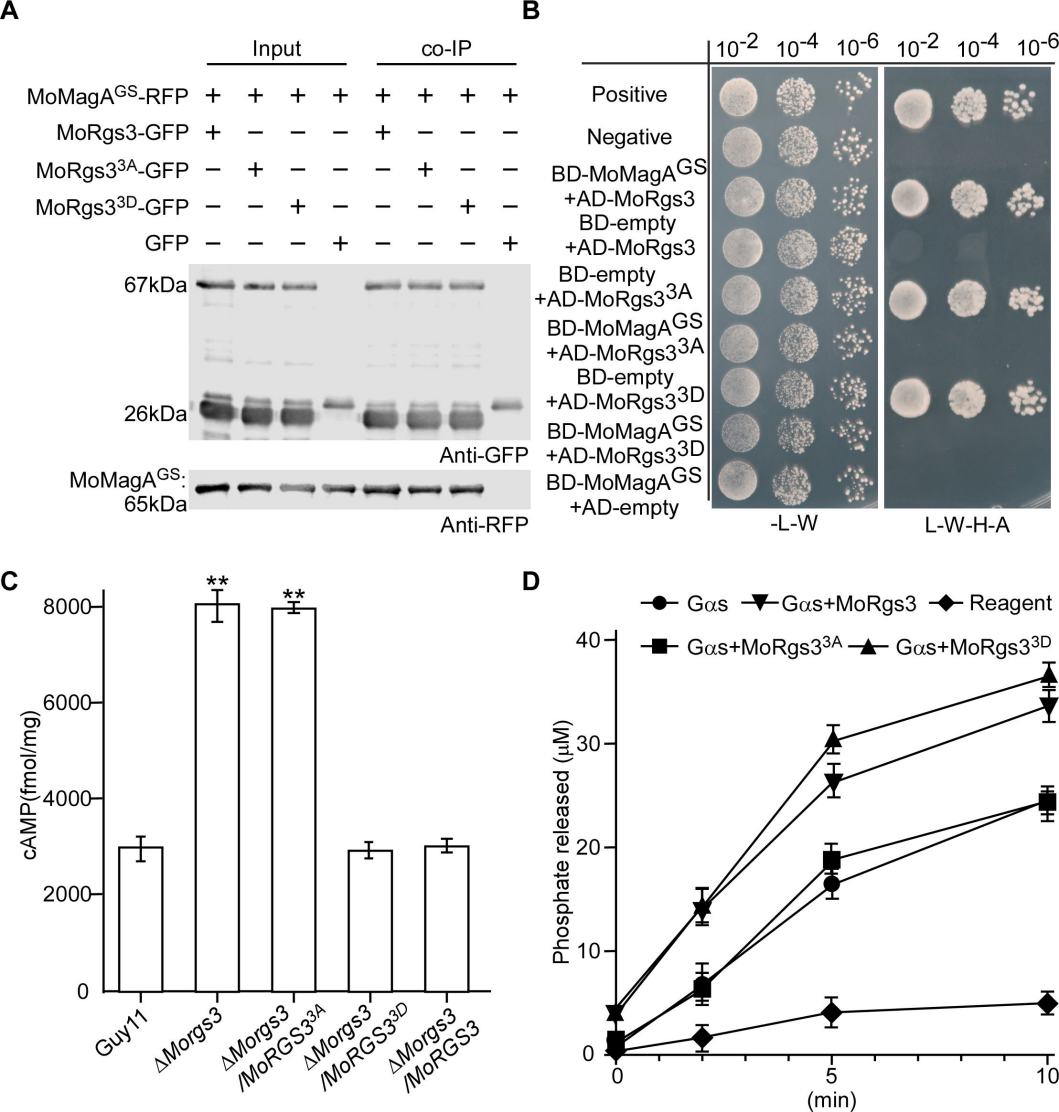
MoNdk1-dependent MoRgs3 phosphorylation is required for MoMagA-cAMP signaling. (**A**) Co-IP assays for the interaction between all three states of MoRgs3 (MoRgs3, MoRgs3^3A^, and MoRgs3^3D^)-GFP with MoMagA^G187S^-RFP transformants were introduced into empty-GFP as a control. Total proteins were extracted individually as the total proteins, eluted from the anti-RFP agarose beads, and analyzed by western blot with the anti-GFP or anti-RFP antibodies. (**B**) Yeast two-hybrid analysis. MoMagA^G187S^ (activated Gα) was co-introduced with MoRgs3 and its site-directed mutagenesis MoRgs3^3A^ and MoRgs3^3D^ into yeast AH109 strain, respectively. Transformants were plated on SD-Leu-Trp and on SD-Leu-Trp-His-Ade for 5 days. (**C**) Measurement of intracellular cAMP levels in mycelia. The levels of cAMP following 2 days of culturing in the complete medium (CM) are shown. The data are evaluated by HPLC analysis with three replicates. Error bars represent SD, and asterisks represent significant differences (*P < 0.05). (**D**) Measurement of GTPase rates. Free phosphates liberated by enzymes were measured using a GTPase activity kit. MoRgs3 and its site-directed mutagenesis MoRgs3^3A^ and MoRgs3^3D^ were measured at least three times. Error bars indicate SDs.

Moreover, we found that MoRgs3^3D^-GFP fluorescence is enhanced at the PM of the Δ*Mocrn1* mutant, similar to MoRgs3-GFP in the Δ*Mocrn1* mutant but in contrast to MoRgs3^3D^-GFP in Guy11. The constitutively phosphorylated MoRgs3^3D^-GFP in Guy11 showed more concentrated fluorescent signals in endosomes than the normal MoRgs3-GFP in Guy11 (Fig. 5E). We also detected the phosphorylation of MoRgs3 in the Δ*Mocrn1* strain and found that MoCrn1 did not affect the phosphorylation of MoRgs3 (Fig. S6E ). The above results indicated that MoRgs3 phosphorylation enhances the bind of MoRgs3 to MoCrn1, supporting that MoCrn1 also regulates MoRgs3 internalization.

### MoRgs3 is involved in MoMagA-mediated cAMP signaling and normal appressorium induction

MoRgs3 is one of the RGS family proteins that also plays an indispensable role in cAMP signaling of *M. oryzae* (19, 23). To further explore MoRgs3 functional mechanisms, we evaluated the *in vitro* GAP activity by measuring levels of free phosphate release using an ATPase/GTPase activity assay kit. The phosphorylation-dead mutant MoRgs3^3A^ lost its GAP function in comparison to MoRgs3 and the mimic phosphomimic MoRgs3^3D^ (Fig. 6D). Since MoRgs3 GAP function is critical for maintaining the intracellular cAMP levels in *M. oryzae* (19, 23), we tested cAMP levels in Δ*Morgs3* and site-directed mutagenesis transformants by HPLC. Higher intracellular cAMP levels were detected in Δ*Morgs3* and Δ*Morgs3/MoRGS3^3A^* than in Guy11 and the Δ*Morgs3/MoRGS3^3D^* complemented strain, indicating MoRgs3 phosphorylation is required for its GAP function (Fig. 6C).

MoMagA is one of the three Gα subunits (MoMagA, MoMagB, and MoMagC) in *M. oryzae*, and it plays a major role in the cAMP pathway (19, 48, 49). We tested interactions of GDP-bound MoMagA with MoRgs3, MoRgs3^3A^, and MoRgs3^3D^ by Y2H, pull-down, and co-IP assays. The results showed that MoRgs3, MoRgs3^3A^, but not MoRgs3^3D^, interact with MoMagA (Fig. S7B through D ), indicating that MoRgs3 phosphorylation results in its dissociation from MoMagA. In addition, we generated BD-MoMagA^G187S^- and MoMagA^G187S^-His constructs that mimic GTP-bound MoMagA to examine if changes in GTPase accelerating protein activities involve changes in binding affinity between GTP-bound MoMagA and MoRgs3. Y2H, pull-down, and *in vivo* co-IP all showed that MoMagA^G187S^ binds to MoRgs3 and MoRgs3^3D^ (Fig. 6A and B). In summary, MoRgs3 phosphorylation enhances cAMP signaling by regulating MoMagA through its GAP function in *M. oryzae*.

## DISCUSSION

*M. oryzae* is the causal agent of rice blast, infecting its host by forming the appressorium that penetrates the host cell (35, 45, 46). G-Protein/cAMP signaling is required for appressorium formation and pathogenicity (23, 50, 51). The regulators of G-protein signaling also play an important role in these function (21). Our studies revealed the role of MoRgs3 in response to intracellular ROS signaling and its regulatory function in cAMP signaling through MoCrn1-mediated endocytosis. Furthermore, our findings indicated that MoNdk1 phosphorylates MoRgs3 in response to intracellular ROS signaling that facilitates its endocytosis. Notably, MoNdk1-dependent phosphorylation of MoRgs3 and subsequent endocytosis are critical for the appressorium development and pathogenicity of *M. oryzae*. These insights contributed to a deeper understanding of the molecular mechanisms underlying the regulation of appressorium formation and pathogenicity by RGS- and RGS-like proteins.

RGS and RGS-like proteins are directly linked to the Gα-cAMP signaling pathway, which works as negative regulators to enhance intrinsic GTPase activities of GTP-bound Gα subunits, thereby inactivating G protein function (52, 53). Various studies explore the unique function of RGS proteins by also focusing on their phosphorylation. In *M. oryzae*, the phosphorylation of RGS proteins plays a significant role in fungal development and pathogenicity. For example, MoRgs1 phosphorylation by the casein kinase 2 MoCk2 is required for appressorium formation and pathogenicity (20). MoRgs7 is phosphorylated by the cell cycle-related kinase MoSep1 in response to hydrophobic surface cues, and the phosphorylation and endocytic transport link signaling transmission to pathogenicity (22). Our current studies provided compelling evidence to indicate that MoRgs3 is phosphorylated by the nucleoside diphosphate kinase MoNdk1.

Phosphorylation is an important cellular regulatory mechanism that plays an important role in the appressorium formation of *M. oryzae* (54). Recent studies have indicated that phosphorylation residues identified in *M. oryzae* are conserved across various filamentous fungi. Thus, identifying phosphorylation sites is important in understanding protein function. In this context, we identified three MoNdk1-dependent phosphorylation sites on MoRgs3 (S93, S134, and S140). Each phosphorylation site may regulate different functions, cellular or/and pathogenicity. For example, previous studies revealed that MoPmk1 phosphorylates MoVts1, MoVts1 is a component of the MoPmk1 MAPK pathway that has two MoPmK1-dependent phosphorylation sites, S175 and S420. Only S175 plays an important role for MoVts1 in appressorium development, and this role is conserved in all strains examined (55). Autophagy-associated protein MoAtg9-dependent phosphorylation of S122 and MoAtg13-dependent phosphorylation of S517 and S519 regulate MoVts1-mediated autophagy (55, 56). Moreover, multiple phosphorylation sites often work together to regulate the phosphorylation process. Conversely, a single point mutation has little effect on protein function, such as in the case of the transcription factor MoHox7-dependent phosphorylation of MoPmk1 (5). MoMkk1 phosphorylation of MoAtg4 was not inhibited by single site-inactivating mutation (57). Among the RGS family proteins, mutations in the single phosphorylation site of MoRgs1 or MoRgs7 will affect the interaction with their kinases, leading to the defects on phosphorylation and pathogenicity (20, 22).

Most of the RGS proteins in *M. oryzae* regulate G protein activity by affecting GAP function, intracellular cAMP levels, and appressorium formation and pathogenicity. These RGS proteins may function differently in the perception of external signals. MoRgs7 contains a C-terminal GPCR motif, in addition to its RGS-like domain, and studies showed that MoRgs7 senses hydrophobic surface cues and regulates Gα MoMagA function in appressorium formation and pathogenicity (22). We examined whether MoRgs3 has a similar response but found that it did not undergo phosphorylation in response to these signals. Notably, MoRgs3 has only three transmembrane domains, unlike MoRgs7 that has seven. Our studies provided evidence that MoRgs3 responds to intracellular ROS signals in its regulation of the cAMP signaling pathway affecting appressorium formation and pathogenicity.

ROS is produced in the metabolic processes of *M. oryzae* and acts as signals in a variety of developmental pathways, including the formation of infection structures. During appressorium development, *M. oryzae* accumulates high levels of endogenous ROS in the tips of its germ tubes and immature appressoria (35, 58, 59). The scavenging of ROS delays the differentiation of the appressorium and alters its morphology (35). Therefore, it is very important to maintain the homeostasis of intracellular ROS levels in *M. oryzae*. Our studies also linked intracellular ROS to MoNdk1 that phosphorylates MoRgs3 and promotes MoRgs3-MoCrn1 binding.

Appressorium formation on nutrient-free, hydrophobic surfaces relies on normal cAMP and MAPK signaling. Without intracellular ROS production, MoRgs3 remains unphosphorylated, impeding cAMP signaling. Consequently, ROS appears as a pivotal regulation factor in the growth and development of *M. oryzae*. The NOx complex is the primary source of ROS generation. It facilitates electron transfer from NADPH to the PM and catalyzes the reduction of O_2_ to produce superoxide O_2_. Superoxide O_2_ is then converted to H_2_O_2_ by the superoxide dismutase to constitute one of the critical regulators in cell growth and differentiation. Our studies revealed the importance and mechanisms of the endogenous ROS as a signaling molecule in regulating the differentiation and pathogenicity of *M. oryzae*.

The *M. oryzae* MoNdk1 reversibly transfers phosphate groups from tri- to diphosphate nucleosides. MoNdk1 mainly phosphorylates at the substrate level, converting GDP to GTP and directing GTP to proteins required by GTP, such as kinetic protein-like GTP enzymes and heterotrimer G proteins (44). Elevated intracellular GDP content disrupts cellular metabolism, disturbs redox balance, and triggers an increase in intracellular ROS concentration, prompting MoNdk1 to phosphorylate MoRgs3. This phosphorylated MoRgs3, in a reciprocal manner, expedites the GTPase activity of the Gα subunit and subsequent GTP hydrolysis. The phosphorylated MoRgs3, in turn, accelerates the GTPase activity of the Gα subunit and the subsequent GTP hydrolysis process. Therefore, we infer that the interaction between MoNdk1 and MoRgs3 serves to maintain intracellular energy and redox balance. This, in turn, mitigates host oxidative burst and suppresses rice innate immunity.

**Fig 7:**
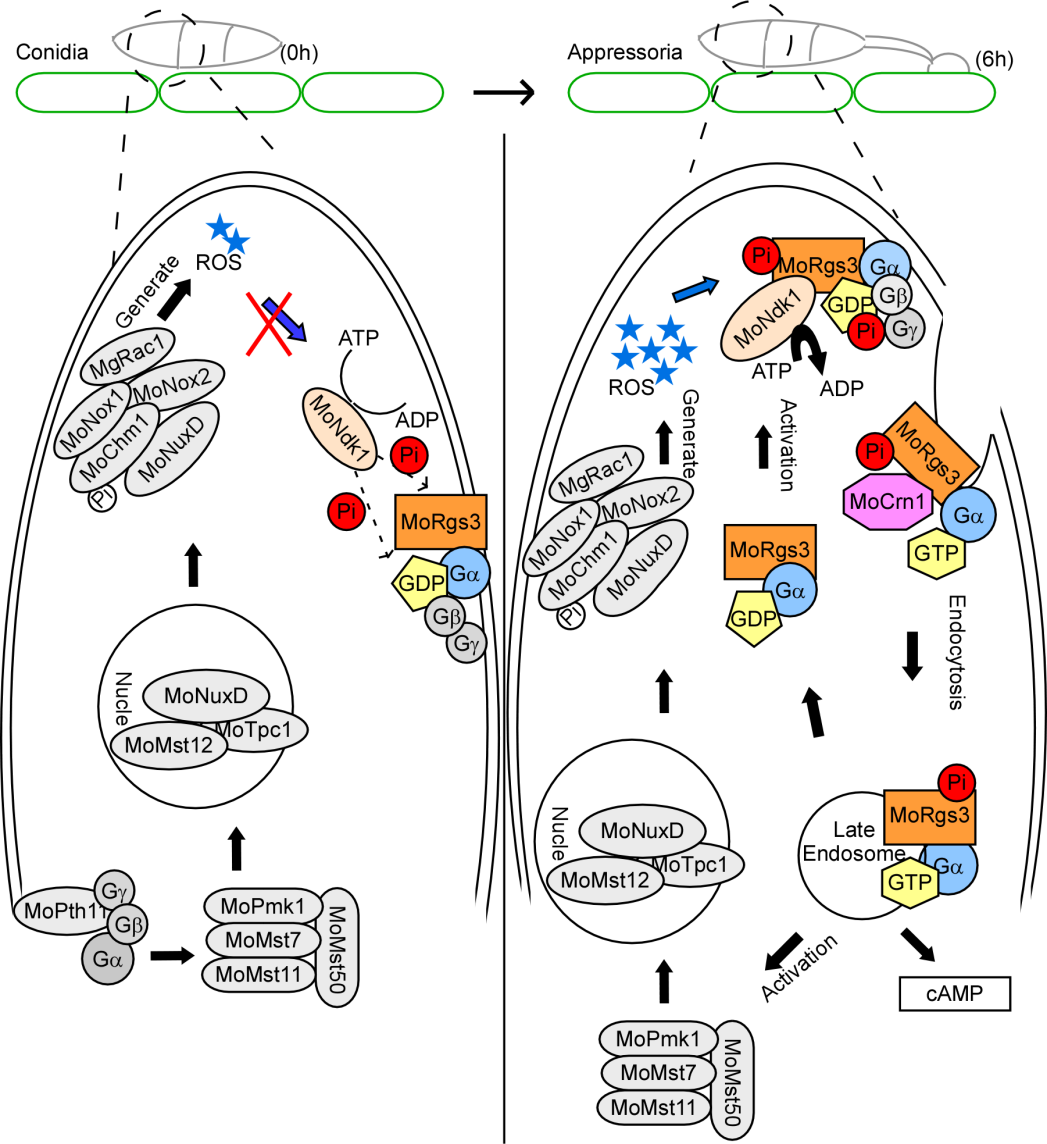
A schematic summary of MoRgs3 function during appressorium formation in *M. oryzae*. Conidial germination of *M. oryzae* was accompanied by a burst of ROS. The low level of intracellular ROS in the conidial stage cannot activate MoRgs3 phosphorylation. Intracellular ROS burst in appressorium stage. MoNdk1 sense the intracellular ROS signal to phosphorylate MoRgs3. The phosphorylated MoRgs3 accelerates the binding to MoCrn1 for endocytosis and then goes into the intracellular to regulate MoMagA-mediated cAMP signal and regulate the formation of appressorium.

In summary, we have provided evidence demonstrating that MoRgs3 functions in regulating appressorium formation and pathogenicity of *M. oryzae* by sensing intracellular ROS levels. MoRgs3 becomes phosphorylated by MoNdk1 in response to increased intracellular ROS levels. Phosphorylated MoRgs3 likely undergoes MoCrn1-dependent endocytosis in a pattern similar to MoRgs1 and MoRgs7. Together, these RGS- and RGS-like proteins respond to different external cues to modulate MoMagA-mediated cAMP signaling and impact appressorium formation and pathogenicity (Fig. 7).

## MATERIALS AND METHODS

The methods are adapted from our previous work for this study. A description of the materials and methods is provided below for reference.

### Strains and cultural conditions

Guy11 was used as the wild-type strain of *M. oryzae* and cultured on a CM. For vegetative growth, small mycelium blocks from 5-day-old colonies were transferred into fresh media and incubated in the dark at 28°C (60). Mycelia were harvested from liquid CM medium grown for 2 days and used for DNA and protein extractions (61, 62). The wild-type and mutants were cultured on an SDC medium at 28°C for 3 days in the dark and transferred to continuous illumination under fluorescent light before being scraped for surface hyphae and conidia evaluation (63, 64).

### Protein extraction and western blot analysis

For total protein extraction, strains were incubated in liquid CM media with shaking for 2 days and harvested. Mycelia were grounded into fine powder in liquid nitrogen and suspended in lysis buffer (10 mM Tris-HCl, pH 7.5, 150 mM NaCl, 0.5 mM EDTA, 0.5% NP-40, and 2 mM PMSF) (65). The lysates were collected into 2.0-mL tubes on ice for 30 min and shaken every 10 min. Lysates were then centrifuged at 15,000 rpm for 10 min at 4°C, and supernatants were collected as total protein extracts (66, 67). For GFP-tagged protein detection, samples were analyzed with 12% SDS-PAGE gel and immunoblotted with anti-GFP antibodies (mouse, 1:5,000, Abmart, 293967). Signals were detected by the ODYSSEY infrared imaging system (software version 2.1).

### Phosphorylation analysis

For *in vitro* analysis, GST-MoRgs3, GST-MoRgs3^3A^, and HIS-MoNdk1 were expressed in *E. coli* DE3 cells and purified (68). We used the Pro-Q Diamond Phosphorylation gel stain (Thermo Fisher Scientific), a phosphor-protein gel-staining fluorescence dye in this assay. A kinase reaction buffer (100 mM phosphate-buffered saline, pH 7.5, 10 mM MgCl_2_, 1 mM ascorbic acid) was mixed with MoRgs3 and MoRgs3^3A^ and HIS-MoNdk1, respectively. The subsequent experiments were performed according to the previously described protocol (20).

For *in vivo* analysis, conidia were prepared from various transformants as described above and were filtered through three layers of lens paper before resuspending in sterile water (2 × 10^5^ spores/mL) (42). For appressorium protein extraction, droplets (5 mL) of spore suspensions were placed on strips of onion epidermis, incubated under humid conditions at room temperature for 6 hours, and onion epidermis grounded for protein extraction (43). Protein extraction was the same as described above, and phosphorylation analysis was performed according to the protocol, phosphatase inhibitors (P0044, sigma), and alkaline phosphatase (P6774, sigma) (22).

### Virulence test

Rice seedlings (*Oryza sativa* cv. CO39) were used for the pathogenicity test. Two-week-old rice seedlings were sprayed with 5 mL of conidia suspension and kept in darkness for 24 hours. Then, rice seedlings were transferred into a transparent growth chamber following a 12-hour/12-hour light/dark cycle exposure schedule. After 7 days, leaf disease severity was documented by photography. Lesions were quantitated by quantitative real-time PCR (69, 70).

Three-week-old rice sheaths were collected and injected with conidia suspensions (2 × 10^5^ spores/mL) with syringes. After 36 hours post-incubation in the dark at 28°C, epidermal cells were observed by microscopy. This experiment was conducted with three biological repetitions and three replicates under the same experimental conditions (45).

### LC-MS-MS analysis and GTPase activity assays

Fungal proteins were extracted as described above and separated by 10% SDS-PAGE gel electrophoresis. Phosphorylation site identification by LC-MS-MS was carried out as described previously (66).

His-MoRgs3, His-MoRgs3^3A^, His-MoRgs3^3D^, and His-MoMagA^G187S^ were expressed in *E. coli* DE3 cells and purified. An ATPase/GTPase activity assay kit (MAK113; Sigma-Aldrich; Merck, Darmstadt, Germany) was used to assess the GTPase activity. The phosphate standards included in the kit were used to plot the standard curve. Reactions were incubated for 0 s, 1 min, 5 min, and 10 min at room temperature. Reagent buffer (200 mL) was added to each well, and incubation was carried out for an additional 30 mins at room temperature to terminate the enzyme reaction and generate the colorimetric end product. Absorbance at 620 nm was read, and the change in absorbance values (DA620) was calculated by subtracting the control well (A620) from the sample well (A620). The concentration (μM) of free phosphate [Pi] was computed in the sample from the standard curve. The relevant experimental procedures were as described previously (20).

### Intracellular cAMP level measurement

*M. oryzae* strains and transformants were cultured in liquid CM for 48 hours and harvested. Mycelia were grounded into fine powders in liquid nitrogen, and total cAMP levels were quantified by HPLC following previously established procedures (20, 71)

### Microscale thermophoresis assay

Bindings of MoCrn1 to MoRgs3, MoRgs3^3A^, MoRgs3^3D^, and MagA^G187S^ to MoRgs3, MoRgs3^3A^, and MoRgs3^3D^ were determined by MST using Monolith NT.115 (Nano Temper Technologies) according to the manufacturer-provided protocol. MST premium-coated capillaries (Monolith NT.115 MO-K005) were used to load the samples into the MST instrument at 25°C using medium MST power. Laser on and off was set at 30 and 5 s, respectively. This experiment was repeated with three biological repetitions and three replicates under the same experimental conditions. Data were analyzed using Nano Temper Analysis software v.1.2.101 (Nano Temper Technologies) (3, 72).

### GST pull-down assay

GST-MoRgs3, His-MoNdk1, and HIS were expressed in *E. coli* BL21-CodonPlus (DE3) cells. Samples were induced with 0.1 mM IPTG (isopropyl-b-D-1-thiogalactopyranoside) at 20°C for 4 hours. *E. coli* cells were lysed in lysis buffer [50 mM Tris (pH = 8.0), 50 mM NaCl, 1 mM PMSF (Sigma Aldrich)]. Samples were centrifuged at 3,600 rpm for 10 min, and the supernatants were transferred to new 1.5-mL tubes and stored at −70°C. The GST-MoRgs3 supernatant was mixed with 30 mL of GST Sepharose beads, incubated at 4°C for 4 hours, and then incubated with HIS-MoNdk1 and HIS supernatants at 4°C for another 4 hours. Finally, the beads were washed with buffer (50 mM Tris, pH 8.0, 50 mM NaCl, 1 mM PMSF, 1% Triton X-100) five times and proteins were eluted. Eluted proteins were analyzed by immunoblot with anti-HIS and anti-GST antibodies (57, 64).

### Confocal laser scanning microscopy

All transformants were observed under a confocal laser scanning microscope (Zeiss LSM710, 63× oil), and the filtered channels were set as follows: GFP (excitation spectra: 488 nm; emission spectra: 510 nm; intensity of fluorescence: 75%; exposure time: 800 ms; insets highlight areas analyzed by line scan; and bar = 10 µm).

### Appressorium formation

Conidia were harvested from 7-day-old SDC cultures and adjusted to 5 × 10^4^ conidia mL^−1^ in sterile water. Droplets (30 µL) of conidial suspension were applied on coverslips (Fisher Scientific, St. Louis, MO) and incubated under humid conditions at 28°C prior to appressorium formation assessment (73). The turgor pressure of mature appressorium was measured using an incipient cytorrhysis (cell collapse) assay (49).

## Data Availability

The genes from this study can be found in the GenBank database (https://www.ncbi.nlm.nih.gov/protein/) using the following accession numbers: MoNdk1 (MGG_08622), MoRgs3 (MGG_03726), MoCrn1 (MGG_06389), MoTrx2 (MGG_04236), and MoMagA (MGG_01818).

## SUPPLEMENTAL MATERIAL

Text S1 mbio.00996-24-s0001.docx Identification of MoRgs3 binding proteins.

Fig. S1 mbio.00996-24-s0002.tif Changes in intracellular redox balance affect the phosphorylation of MoRgs3 in conidial stage.

Fig. S2 mbio.00996-24-s0003.tif Changes in intracellular redox balance affect the phosphorylation of MoRgs3 in appressorial stage.

Fig. S3 mbio.00996-24-s0004.tif MoNdk1-dependent MoRgs3 phosphorylation sites identified by LC-MS-MS (Q-E) analysis.

Fig. S4 mbio.00996-24-s0005.tif MoNdk1 phosphorylates MoRgs3 by sensing intracellular ROS signals.

Fig. S5 mbio.00996-24-s0006.tif Continuously phosphorylated MoRgs3 could not fully restore the defect of MoNdk1 in growth and virulence.

Fig. S6 mbio.00996-24-s0007.tif MoCrn1 interacts with MoRgs3, MoRgs3^3A^ and MoRgs3^3D^.

Fig. S7 mbio.00996-24-s0008.tif MoMagA interacts with MoRgs3, MoRgs3^3A^, and MoRgs3^3D^.

Legends mbio.00996-24-s0009.doc Legends for supplemental material.

ASM does not own the copyrights to Supplemental Material that may be linked to, or accessed through, an article. The authors have granted ASM a non-exclusive, world-wide license to publish the Supplemental Material files. Please contact the corresponding author directly for reuse.
