# Supplementary material for: MoRgs3 functions in intracellular reactive oxygen species perception-integrated cAMP signaling to promote appressorium formation in Magnaporthe oryzae
Source: mBio. 2024 Jul 9;15(8):e00996-24. doi: 10.1128/mbio.00996-24 (PMC11323498; doi:10.1128/mbio.00996-24)
Supplement: Text S1 — Identification of MoRgs3 binding proteins. [file mbio.00996-24-s0001.docx]

| Gene ID | Predicted function |
| --- | --- |
| MGG_00952 | Amidohydrolase 2 |
| MGG_04564 | A negative regulator of RAS-cAMP pathway in S. cerevisiae |
| MGG_05421 | Uncharacterized protein |
| MGG_06903 | Uncharacterized protein |
| MGG_06689 | The catalytic domain of chitin synthase III |
| MGG_07471 | Consists of metazoan vacuolar ATP synthase subunit S1 proteins |
| MGG_08080 | synthase subunit S1 proteins |
| MGG_08622 | Nucleoside diphosphate kinase |
| MGG_09457 | Thymidylate kinase |
| MGG_09963 | Uncharacterized protein |
| MGG_10604 | A part of the mitochondrial respiratory chain |
| MGG_11496 | Comprised of fungal proteins with multiple transmembrane regions |

**S1 Text. Identification of MoRgs3 binding proteins.** The bait construct BD-MoRgs3 was used to screen a yeast two-hybrid cDNA library constructed with an RNA pool from various stages, including conidia and infectious hyphae (0, 2, 4, 8, 12 and 24 h).
