# Supplementary figures and images for: MoRgs3 functions in intracellular reactive oxygen species perception-integrated cAMP signaling to promote appressorium formation in Magnaporthe oryzae
Source: mBio. 2024 Jul 9;15(8):e00996-24. doi: 10.1128/mbio.00996-24 (PMC11323498; doi:10.1128/mbio.00996-24)

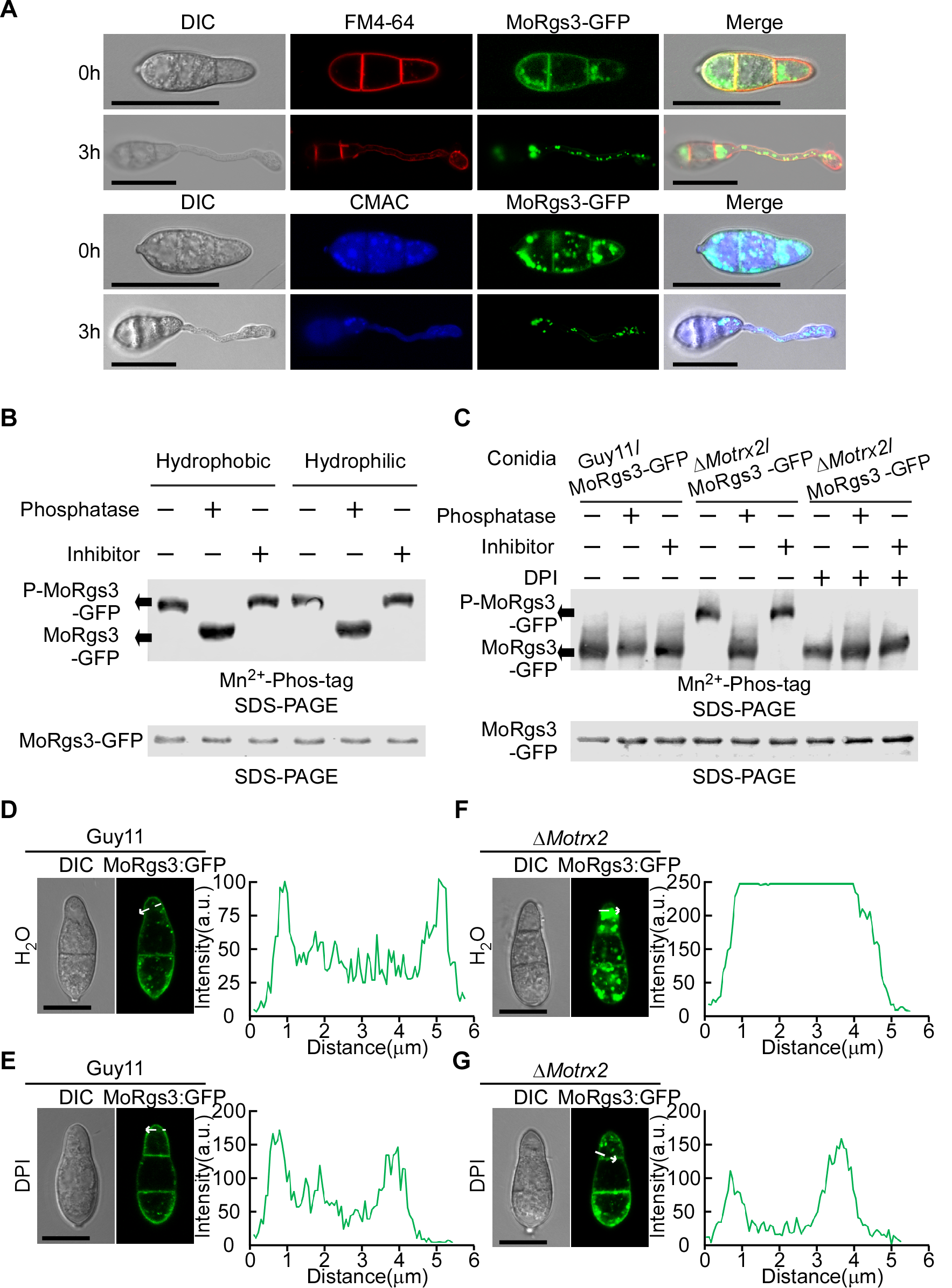

Supplement: Fig. S1 — Changes in intracellular redox balance affect the phosphorylation of MoRgs3 in conidial stage. [file mbio.00996-24-s0002.tif]

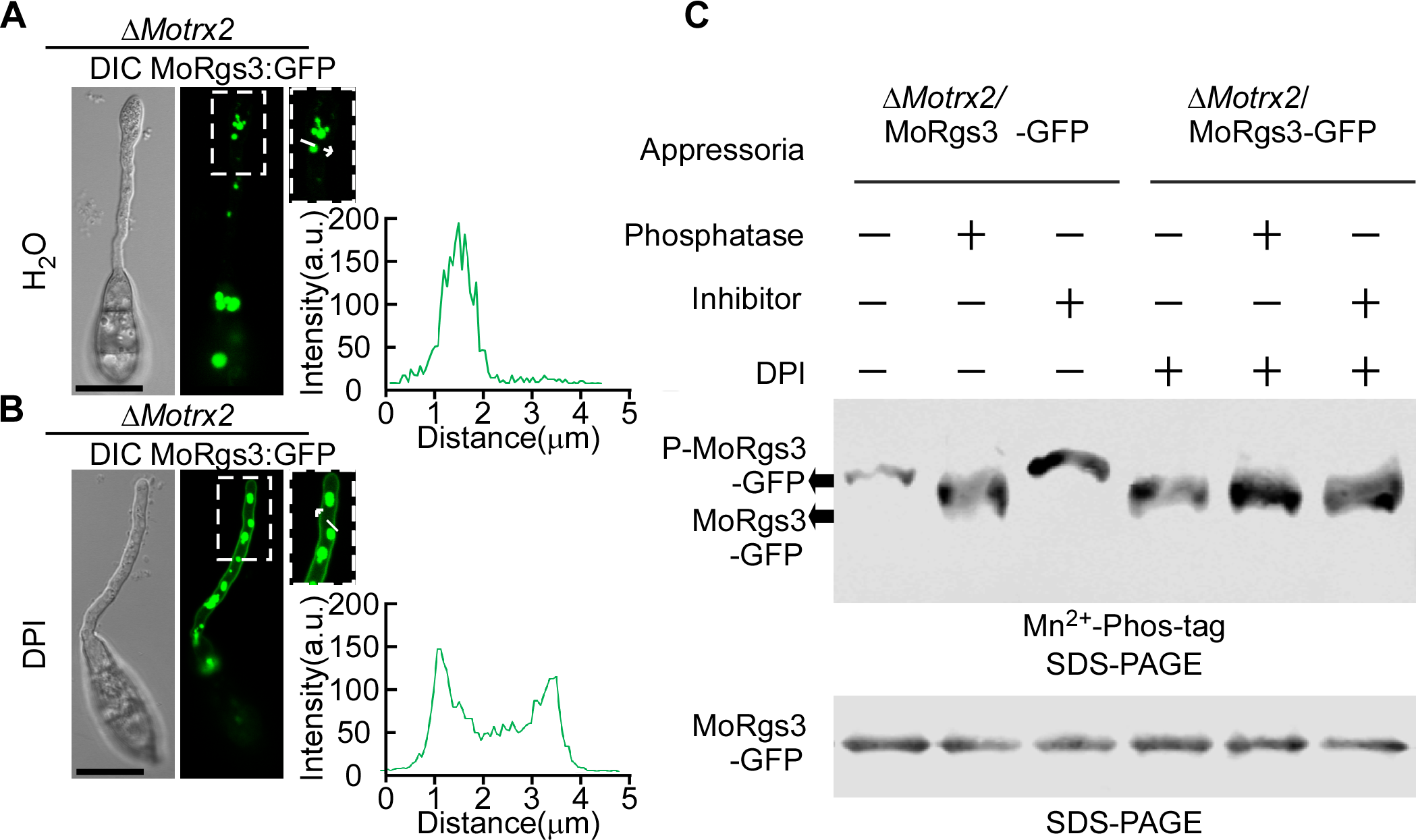

Supplement: Fig. S2 — Changes in intracellular redox balance affect the phosphorylation of MoRgs3 in appressorial stage. [file mbio.00996-24-s0003.tif]

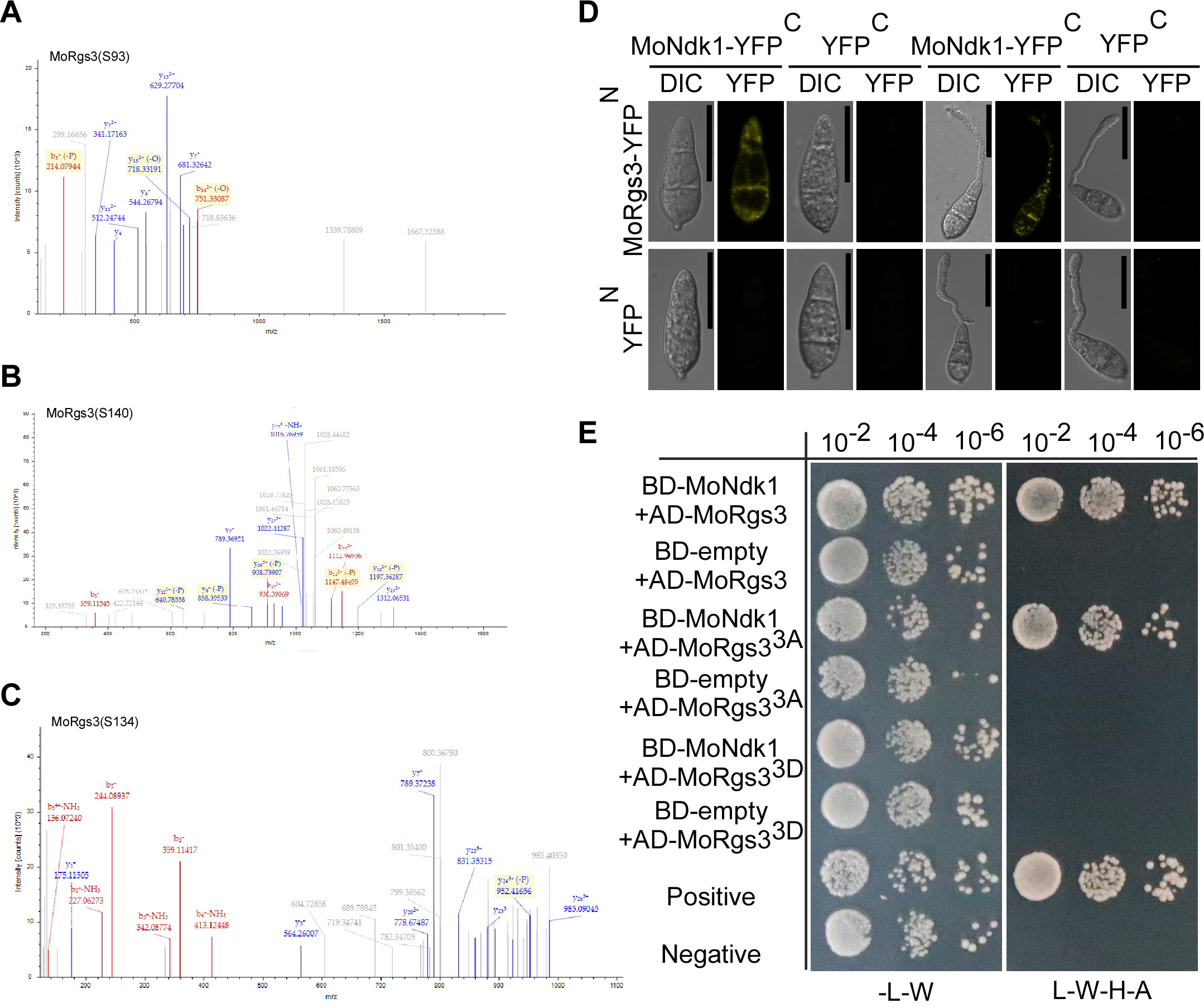

Supplement: Fig. S3 — MoNdk1-dependent MoRgs3 phosphorylation sites identified by LC-MS-MS (Q-E) analysis. [file mbio.00996-24-s0004.tif]

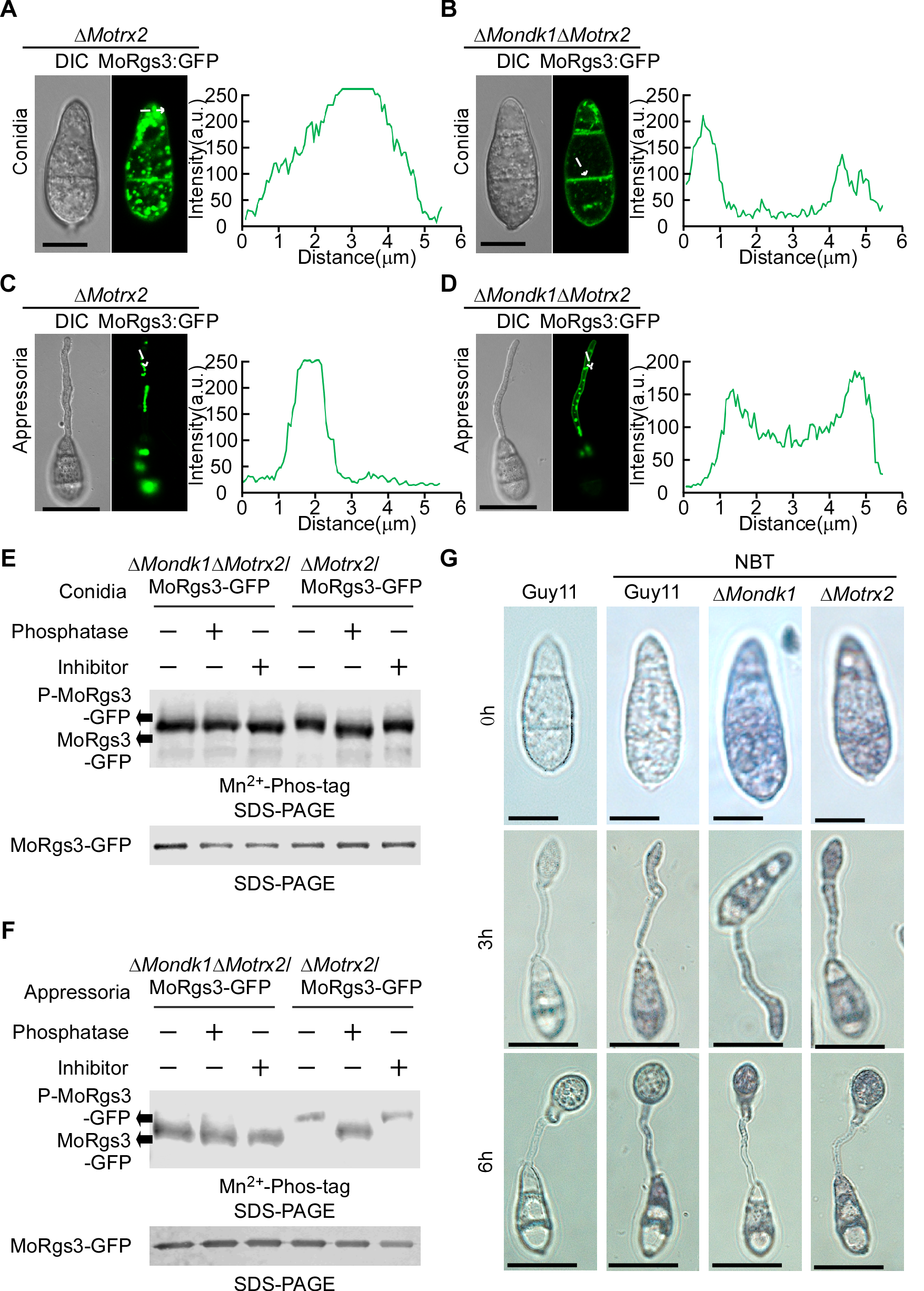

Supplement: Fig. S4 — MoNdk1 phosphorylates MoRgs3 by sensing intracellular ROS signals. [file mbio.00996-24-s0005.tif]

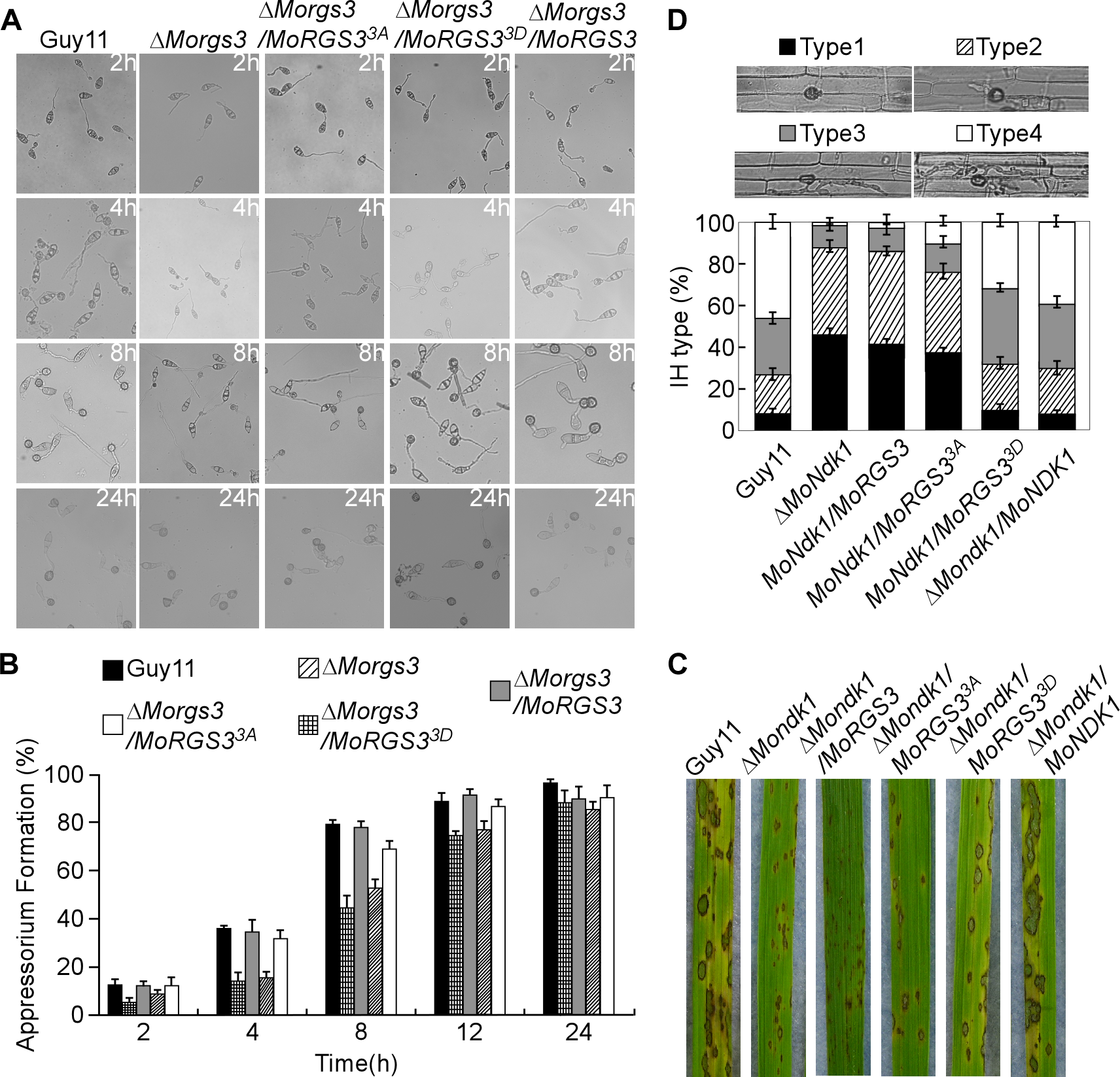

Supplement: Fig. S5 — Continuously phosphorylated MoRgs3 could not fully restore the defect of MoNdk1 in growth and virulence. [file mbio.00996-24-s0006.tif]

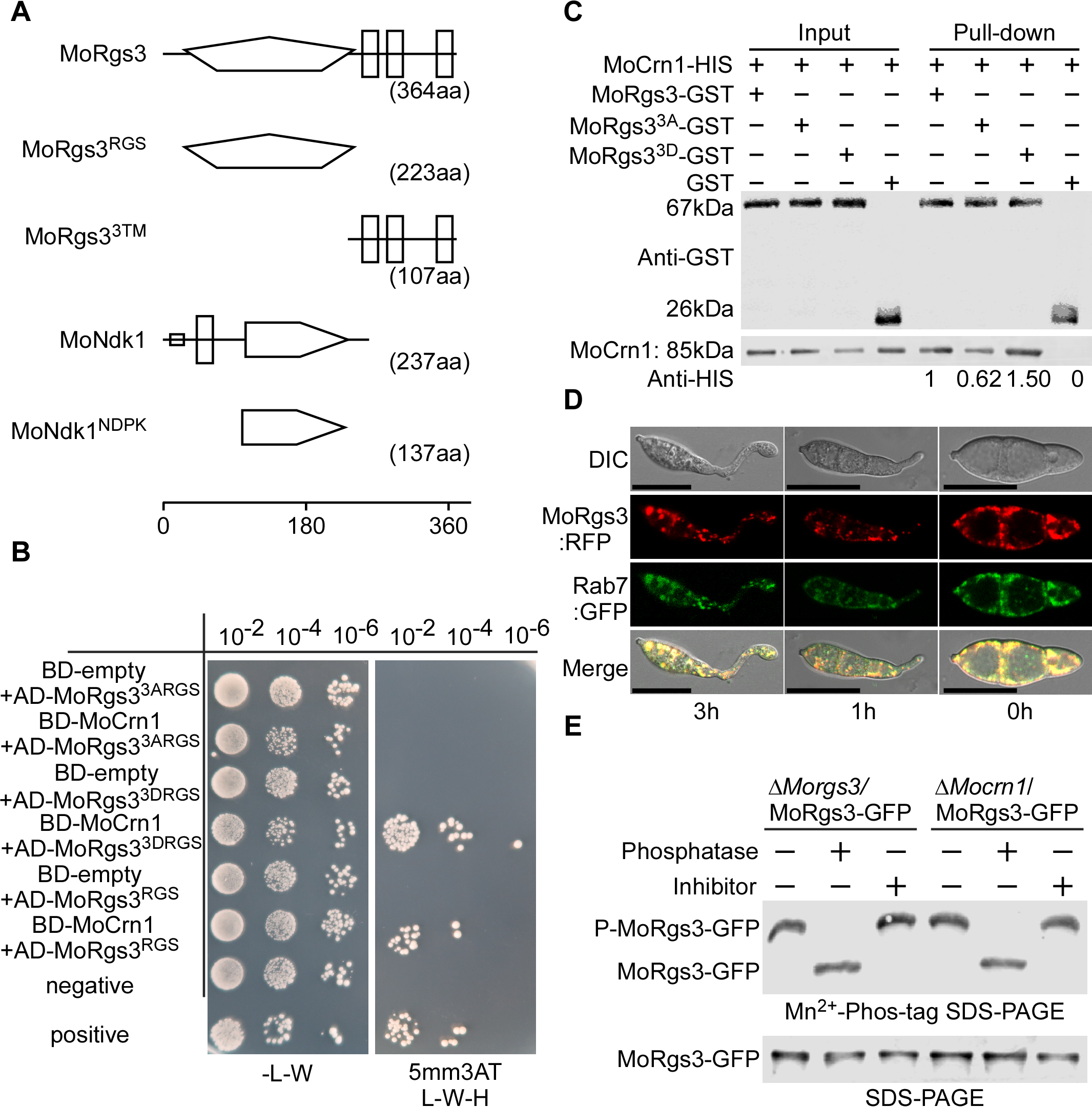

Supplement: Fig. S6 — MoCrn1 interacts with MoRgs3, MoRgs33A and MoRgs33D. [file mbio.00996-24-s0007.tif]

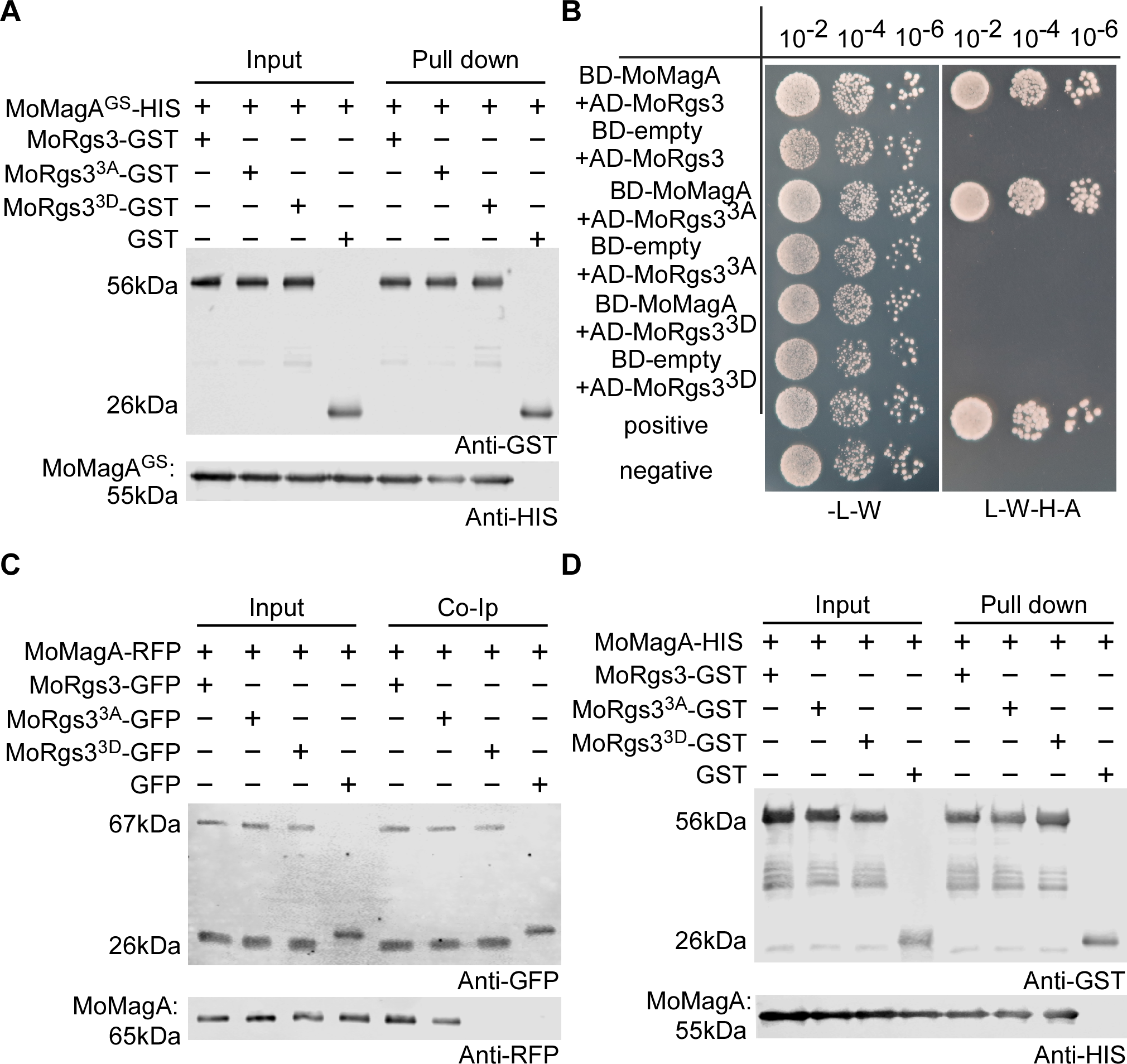

Supplement: Fig. S7 — MoMagA interacts with MoRgs3, MoRgs33A, and MoRgs33D. [file mbio.00996-24-s0008.tif]
