# Supplementary material for: MoRgs3 functions in intracellular reactive oxygen species perception-integrated cAMP signaling to promote appressorium formation in Magnaporthe oryzae
Source: mBio. 2024 Jul 9;15(8):e00996-24. doi: 10.1128/mbio.00996-24 (PMC11323498; doi:10.1128/mbio.00996-24)
Supplement: Legends — for supplemental material. [file mbio.00996-24-s0009.doc]

**Fig. S1. Changes in intracellular redox balance affect MoRgs3 phosphorylation during the conidial stage.**

(A) Plasma membrane dye FM4-64 and vesicle dye CMAC staining experiments. Fluorescence microscopy was used to observe the localization of MoRgs3-GFP after FM4-64 and CMAC staining. (B) Phosphorylation analysis of MoRgs3-GFP in Guy11. MoRgs3-GFP proteins were extracted from conidia of transformants germinated on hydrophobic and hydrophilic surfaces at 4 hours post-inoculations. (C) MoRgs3-GFP proteins were extracted from transformants at WT, Δ*Motrx2* and DPI treated mutant Δ*Motrx2* strain in the conidial stage then treated with phosphatase and phosphatase inhibitors. Mn^2+^-Phos-tag SDS-PAGE and normal SDS-PAGE were used to conduct western blot analysis with the anti-GFP antibody. The extent of MoRgs3 phosphorylation was estimated by the mobility shift assay. (D-G) Fluorescence GFP labeled MoRgs3-GFP fusion constructs were introduced into the WT and Δ*Motrx2* strains at the conidial stage (3 hpi). And DPI was added to inhibit intracellular ROS production, and green fluorescent labeling was observed. Insets highlight areas analyzed by line-scan. Bar = 5 μm. Percentage of a pattern showed in image was calculated by observation for 50 germinated conidia that were randomly chosen, and observation was conducted for 3 times.

**Fig. S2. Changes in intracellular redox balance affect MoRgs3phosphorylation during the appressorial stage.**

(A and B） Fluorescence GFP labeled MoRgs3-GFP fusion constructs were introduced into the WT and Δ*Motrx2* strains at the germ tube hooking stage (3 hpi). And DPI was added to inhibit intracellular ROS production, and green fluorescent labeling was observed. Insets highlight areas analyzed by line-scan. Bar = 5 μm. Percentage of a pattern showed in image was calculated by observation for 50 germinated conidia that were randomly chosen, and observation was conducted for 3 times. （C）MoRgs3-GFP proteins were extracted from transformants at Δ*Motrx2* and DPI treated mutant Δ*Motrx2* strain at the appressorium stage then treated with phosphatase and phosphatase inhibitors. Mn^2+^-Phos-tag SDS-PAGE and normal SDS-PAGE were used to conduct Western blot analysis with the anti-GFP antibody. The extent of MoRgs3 phosphorylation was estimated by the mobility shift assay.

**Fig. S3. MoNdk1-dependent MoRgs3 phosphorylation sites identified by LC-MS-MS (Q-E) analysis.** (A-C) MoRgs3 phosphorylation sites in Guy11 in comparison with the Δ*Mondk1* mutant expressing MoRgs3 variants. (D) The interaction MoNdk1-YFP^C^ with MoRgs3-YFP^N^ were conducted by BiFC. Empty YFP^C^ and empty YFP^N^ constructs were used as a negative control. The co-transformants were observed at the germ tube hooking stage (3 h) with laser scanning microscopy (Zeiss LSM710 laser scanning microscope, 63 × oil). (E) Yeast two-hybrid analysis. MoNdk1 was co-introduced with MoRgs3 and the site-directed mutagenesis mutants MoRgs3^3A^ and MoRgs3^3D^ into the AH109 strain. The transformants were plated on SD-Leu-Trp (as control), SD-His-Leu-Trp (for initial selection), and SD-Leu-Trp-His-Ade (for further selection) for 5 days.

**Fig. S4. MoNdk1 phosphorylates MoRgs3 by sensing intracellular ROS signals.** (A-D) Images show the MoRgs3-GFP distributions in Δ*Motrx2* and Δ*Mondk1*/Δ*Motrx2* in conidia, and appressorium stages. White arrows indicated the regions where the fluorescence intensity was measured by line-scan analysis. Percentage of a pattern showed in image was calculated by observation for 50 germinated conidia that were randomly chosen, and observation was conducted for 3 times. Bar = 5 μm. (E and F) Analysis of MoRgs3 phosphorylation in Δ*Motrx2* and Δ*Mondk1*/Δ*Motrx2* at the conidial and appressorium stages. (G) Detection of superoxide by NBT staining during germination and appressorium formation in *M. oryzae* strain Guy11, Δ*Mondk1* and Δ*Motrx2* conidia were inoculated on glass coverslips and incubated in a moist chamber at 26°C for 0, 3, and 6 h before being stained with a 0.3 mM NBT aqueous solution for 20 min and viewed by bright-field microscopy.

**Fig. S5. Continuously phosphorylated MoRgs3 could not fully restore the defect of MoNdk1 in growth and virulence**. (A and B) Appressorium formation assays and statistics analysis. Conidia of the WT, Δ*MoRgs3*, Δ*MoRgs3*/*MoRgs3^3A^*, Δ*MoRgs3*/*MoRgs3^3D^* and complemented *MoRGS3* (Δ*MoRgs3*/*MoRgs3*) strains were dropped on hydrophobic surfaces and the dynamics of appressorium formation were photographed at various times (***p* < 0.01, n = 100). Bar = 5 μm. (C and D) Pathogenicity assay, diseased leaf area analysis, and infectious hyphal type assessment were conducted as the same as described for Fig 4.

**Fig. S6. MoCrn1 interaction with MoRgs3, MoRgs3^3A^, and MoRgs3^3D^.** (A) Schematic representation of MoRgs3 and MoNdk1.The RGS domain and NDPK domain were predicted by the SMART program (http//smart.embl-heidelberg.de/). (B) Y2H assays for examining interactions between AD-MoRgs3^RGS^, AD-MoRgs3^3ARGS^, AD-MoRgs3^3DRGS^ and BD-MoCrn1. Yeast co-transformants expressing the bait and prey constructs were isolated on SD-Leu-Trp plate for 3 days and screened by culturing on SD-His-Leu-Trp plates containing 5mm 3AT for 5 days. (C) The interaction GST-MoRgs3, GST-MoRgs3^3A^, GST-MoRgs3^3D^ with His-MoCrn1 were conducted by GST pull-down assays. Image J was used to analyze and compare the gray values of GST pull-down. (D) Late endosome marker GFP-MoRab7 was co-transformed with MoRgs3-RFP in WT strains and observed at conidia and appressorium hooking stage (3h). All assays were observed over 100 samples and insets highlight areas analyzed by line-scan. Bar = 5 μm. (E) Phosphorylation analysis of MoRgs3 *in vivo* by Mn^2+^-Phos-tag gel. MoRgs3-GFP was transferred into Δ*Morgs3* and Δ*Mocrn1* strains, MoRgs3 protein was extracted, and the phosphatase and phosphatase inhibitor treated MoRgs3-GFP protein was detected by GFP antibody, and the Mn^2+^-Phos-tag SDS-PAGE and normal SDS-PAGE were displaced, respectively.

**Fig. S7. MoMagA interaction with MoRgs3, MoRgs3^3A^, and MoRgs3^3D^.** (A) The interaction GST-MoRgs3, GST-MoRgs3^3A^, GST-MoRgs3^3D^ with His- MoMagA^G187S^ (activated Gα) were conducted by GST pull-down assays. GST-MoRgs3, GST-MoRgs3^3A^, GST-MoRgs3^3D^, His- MoMagA^G187S^, and GST were expressed and purified by affinity chromatography. Bound proteins were separated by SDS-PAGE in duplicate and analyzed by Western blot with the anti-HIS and anti-GST antibodies. (B) Yeast two-hybrid analysis. MoMagA was co-introduced with MoRgs3 and the site-directed mutagenesis mutants MoRgs3^3A^ and MoRgs3^3D^ into the AH109 strain. The transformants were plated on SD-Leu-Trp (as control), SD-His-Leu-Trp (for initial selection), and SD-Leu-Trp-His-Ade (for further selection) for 5 days. (C) Co-IP assay for the interaction between MoMagA, MoRgs3 and its site-directed mutagenesis MoRgs3^3A^ and MoRgs3^3D^. Co-expression of MoMagA-RFP and MoRgs3-GFP, MoRgs3^3A^-GFP and MoRgs3^3D^-GFP in Guy11, respectively. Proteins were incubated with anti-GFP beads and detected by anti-GFP and anti-RFP antibodies. (D) The interaction among all three states of MoRgs3 (GST-MoRgs3, GST-MoRgs3^3A^, and GST-MoRgs3^3D^) with His-MoMagA were conducted by GST pull-down assays. GST-MoRgs3, GST-MoRgs3^3A^, and GST-MoRgs3^3D^, His-MoMagA, and GST were expressed and purified by affinity chromatography. Bound proteins were separated by SDS-PAGE in duplicate and analyzed by Western blot with the anti-HIS (Mouse; M20001; Abmart) and anti-GST antibodies (Mouse; M20007; Abmart).

**Text S1. Identification of MoRgs3 binding proteins.** The bait construct BD-MoRgs3 was used to screen a yeast two-hybrid cDNA library constructed with an RNA pool from various stages, including conidia and infectious hyphae (0, 2, 4, 8, 12 and 24 h).
